# Evidence for Dynamic Network Regulation of *Drosophila* Photoreceptor Function from Mutants Lacking the Neurotransmitter Histamine

**DOI:** 10.3389/fncir.2016.00019

**Published:** 2016-03-22

**Authors:** An Dau, Uwe Friederich, Sidhartha Dongre, Xiaofeng Li, Murali K. Bollepalli, Roger C. Hardie, Mikko Juusola

**Affiliations:** ^1^Department of Biomedical Science, University of SheffieldSheffield, UK; ^2^Department of Physiology Development and Neuroscience, Cambridge UniversityCambridge, UK; ^3^National Key Laboratory of Cognitive Neuroscience and Learning, Beijing Normal UniversityBeijing, China

**Keywords:** visual perception, photoreceptor cells, information theory and signal processing, feedback synapses, histamine

## Abstract

Synaptic feedback from interneurons to photoreceptors can help to optimize visual information flow by balancing its allocation on retinal pathways under changing light conditions. But little is known about how this critical network operation is regulated dynamically. Here, we investigate this question by comparing signaling properties and performance of wild-type *Drosophila* R1–R6 photoreceptors to those of the *hdc*^*JK*910^ mutant, which lacks the neurotransmitter histamine and therefore cannot transmit information to interneurons. Recordings show that *hdc*^*JK*910^ photoreceptors sample similar amounts of information from naturalistic stimulation to wild-type photoreceptors, but this information is packaged in smaller responses, especially under bright illumination. Analyses reveal how these altered dynamics primarily resulted from network overload that affected *hdc*^*JK*910^ photoreceptors in two ways. First, the missing inhibitory histamine input to interneurons almost certainly depolarized them irrevocably, which in turn increased their excitatory feedback to *hdc*^*JK*910^ R1–R6s. This *tonic* excitation depolarized the photoreceptors to artificially high potentials, reducing their operational range. Second, rescuing histamine input to interneurons in *hdc*^*JK*910^ mutant also restored their normal *phasic* feedback modulation to R1–R6s, causing photoreceptor output to accentuate dynamic intensity differences at bright illumination, similar to the wild-type. These results provide mechanistic explanations of how synaptic feedback connections optimize information packaging in photoreceptor output and novel insight into the operation and design of dynamic network regulation of sensory neurons.

## Introduction

An abundance of feedback synapses characterizes the ultrastructure of both invertebrate eyes and vertebrate outer retinae, underlining their importance in parallel image processing (Sterling, 1983; Meinertzhagen and O’Neil, 1991). In the spatial domain, lateral inhibitory feedback, from horizontal cells onto cone and rod outputs, results in antagonistic center-surround receptive fields that accentuate image contrasts (Thoreson et al., 2008; Jackman et al., 2011). Chromatically, negative feedback to cones is deemed critical for color constancy and opponency in non-mammalian vertebrates (Burkhardt, 1993; Thoreson and Mangel, 2012). But in the temporal domain, it is less well understood how interneuron feedback contributes to photoreceptors’ signaling dynamics and performance, partly because acquiring long-lasting intracellular recordings from the intact vertebrate retina is difficult.

**FIGURE 1:**
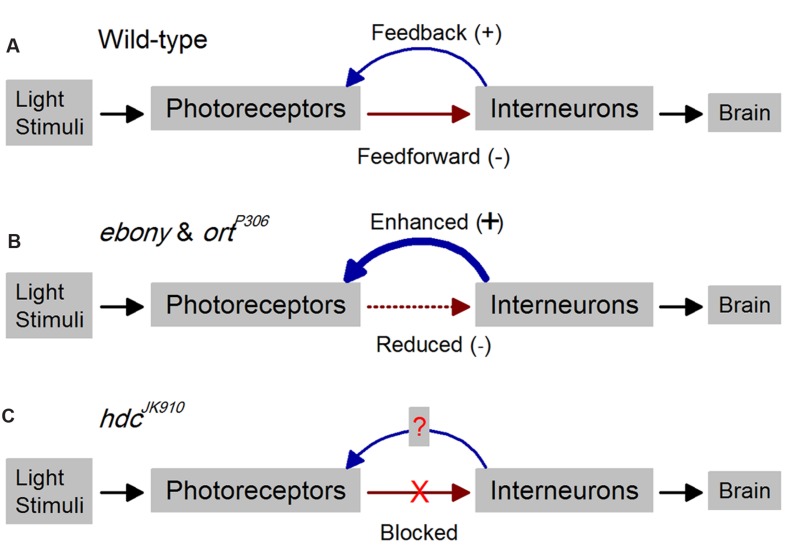
**Outline for studying time-dependent synaptic feedback effects on *Drosophila* photoreceptor functions.** Schematic of R1–R6 photoreceptor-interneuron circuits in wild-type and mutant laminae. **(A)** In the wild-type, inhibitory histaminergic feedforward (-) and excitatory feedback (+) connections are dynamically balanced. **(B)** Reduced inhibitory feedforward synaptic transmissions in *ebony* and *ort*^*P*306^ lead to enhanced excitatory feedback from interneurons to their photoreceptors (Zheng et al., 2006). **(C)** In *hdc*^*JK*910^, the inhibitory feedforward pathway is completely blocked, enabling us to investigate how this affects the interneuron feedback and consequently R1–R6 output.

The *Drosophila* eye is an advantageous model system to study time-dependent feedback functions (**Figure 1**). Its photoreceptors and interneurons encode comparable visual environments to many vertebrate retinae yet are accessible to high-quality intracellular recordings *in vivo*. Synaptic connections in the photoreceptor-lamina network have been reconstructed from electron-micrographs (Rivera-Alba et al., 2011), providing wiring diagrams for local interactions. R1–R6 photoreceptors, which sample light information from the same point in space, form output synapses onto large monopolar cells L1–L3 (LMCs) and amacrine cells (ACs), while most feedback connections to photoreceptors are from L4, L2, and ACs. Histamine is likely the photoreceptors’ sole neurotransmitter, driving the inhibitory feedforward pathway (Hardie, 1987, 1989; Sarthy, 1991), whereas direct feedback elements to photoreceptors seem excitatory; glutamatergic and cholinergic (Kolodziejczyk et al., 2008; Raghu and Borst, 2011; Takemura et al., 2011; Hu et al., 2015). Importantly, fly genetics provide tools to modify transmission in both directions. Our earlier work indicated that interneuron feedback adjusts photoreceptor output actively (**Figure 1A**), protecting it from saturation and improving its signal quality with enriched modulation (Zheng et al., 2006). Findings from mutants revealed that feedforward and feedback are tightly coupled, where defect in one pathway leads to detrimental alteration in the other (**Figure 1B**), resulting in impaired network adaptation (Nikolaev et al., 2009; Zheng et al., 2009) and suboptimal vision (Hu et al., 2015). These results further support studies from blowfly (*Calliphora vicina*) photoreceptors and LMCs, which showed that the communication within the photoreceptor-lamina network is graded and continuous in darkness and light (Laughlin et al., 1987; Juusola et al., 1995; Uusitalo et al., 1995a,b).

Histidine decarboxylase, the enzyme responsible for histamine synthesis, is coded by the *hdc* gene in the *Drosophila* genome (Burg et al., 1993). The null allele *hdc*^*JK*910^ lacks histamine, and its photoreceptors cannot communicate synaptically with interneurons, making these mutants presumably blind. Nonetheless, since its phototransduction appears similar to that in wild-type flies (Gonzalez-Bellido et al., 2009) and its feedback pathways are intact, this mutant can give useful insight into dynamic network regulation (**Figure 1C**).

To investigate how a lack of histamine affects the functional roles of interneuron feedback in shaping photoreceptor output, we examined the signaling dynamics and performance of *hdc*^*JK*910^ R1–R6 photoreceptors in darkness and over a broad light intensity range. We found that the lack of the inhibitory feedforward pathway causes excitatory interneuron feedback to be tonic and enhanced, which in turn depolarizes dark-adapted *hdc*^*JK*910^ photoreceptors to artificially high resting potentials. Hence, the absence of inhibitory (histaminergic) inputs to *hdc*^*JK*910^ LMCs and ACs must depolarize these cells continuously (in darkness and in light) to increase their tonic excitatory load to R1–R6s. In concordance, under prolonged bright stimulation *hdc*^*JK*910^ photoreceptors exhibited smaller responses and narrower operational ranges than the wild-type photoreceptors but near normal adaptation and information transfer. Remarkably, feeding the mutants with histamine rescued their photoreceptor function and visual behavior to the wild-type levels. Our results imply that *hdc*^*JK*910^ photoreceptor output is compressed by tonic excitatory feedback overdrive from interneurons that lacks its normal phasic modulation, and underline the vital role of local interneurons in regulating photoreceptor function and normal vision.

## Materials and Methods

### Fly Stocks

*hdc*^*JK*910^ flies were a gift from Erich Buchner’s lab (Julius-Maximilians-Universität, Würzburg, Germany). As part of stocks maintenance procedures, wild-type and mutant flies were regularly checked by their clearly distinguishable electroretinograms (ERG). *hdc*^*JK*910^ ERGs lack on- and off-transients (Burg et al., 1993; Melzig et al., 1996, 1998), implying that R1–R6 photoreceptor output synapses in the lamina fail to transmit light information to visual interneurons. Note that the inner photoreceptors (R7/R8) are also affected in *hdc*^*JK*910^ mutant, but this effect is not analyzed here; see (Wardill et al., 2012). Flies were reared in standard fly food medium with 12:12 h dark:light cycle and kept at room temperature (20–22°C).

#### Histamine Rescue

Following the published protocol (Melzig et al., 1998), *hdc*^*JK*910^ flies were transferred to a vial containing a Whatman filter soaked in an aqueous 5% histaminediphosphate (Sigma, UK) solution and kept there 24 h before the behavioral and electrophysiological experiments.

### *In Vivo* Electrophysiology

#### Intracellular Recordings

We prepared 3–7 days old (adult) female flies for *in vivo* experiments. A fly was fixed in a conical fly holder with beeswax, and a small hole (6–10 ommatidia) for the recording microelectrode entrance was cut in its dorsal cornea and Vaseline-sealed to protect the eye (Juusola and Hardie, 2001b; Zheng et al., 2006). Conventional filamented sharp quartz and borosilicate microelectrodes (Sutter Instruments, USA), filled with 3 M KCl and having 120–200 MΩ resistance, were used for intracellular recordings from R1–R6 photoreceptors. A reference electrode, filled with fly ringer, was gently pushed through ocelli ∼100 μm into the fly head. Only stable high quality recordings, which lasted tens of minutes without clear changes in sensitivity or resting potential, were included in this study. We have optimized the intracellular recordings method, together with bespoke hardware and software tools, over the last 18 years to provide high-quality long-lasting recordings. Therefore, experienced experimentalists in our laboratory can obtain high-quality penetrations with 60–95% success rate. In darkness, the resting potentials of both wild-type Canton-S and *hdc*^*JK*910^ mutants were <-50 mV and maximum responses to saturating bright pulses were >40 mV. In the experiments, the fly temperature was kept at 19 ± 1°C by a feedback-controlled Peltier device (Juusola and Hardie, 2001b). Note, we could not identify intracellular *hdc*^*JK*910^ LMC penetrations because we did not find their voltage responses to light.

Light stimulation was delivered to the studied R1–R6 photoreceptor at the center of its receptive field with a high-intensity green light-emitting diode (LED) (Marl Optosource, with peak emission at 525 nm), through a fiber optic bundle, fixed on a rotatable Cardan arm, subtending 5° as seen by the fly. Its intensity was controlled by neutral density filters (Kodak Wratten; Juusola and Hardie, 2001b). The results are mostly shown for Dim (∼6,000 photons/s), Medium (Mid: ∼6 × 10^5^ photons/s), and Bright luminance (∼6 × 10^6^ photons/s), as extrapolated from earlier single photon response calibrations; or log -3, log -1, and log 0, respectively.

Voltage responses were amplified in current-clamp mode using a 15 kHz switching rate (SEC-10L single-electrode amplifier; NPI Electronic, Germany). The stimuli and responses were low-pass filtered at 500 Hz (KemoVBF8), and sampled at 1 or 10 kHz. The data were often re-sampled/processed off-line at 1–2 kHz for the analysis. Stimulus generation and data acquisition were performed by custom-written Matlab (MathWorks, USA) programs: BIOSYST (Juusola and Hardie, 2001b; Juusola and de Polavieja, 2003), with an interface package for National Instruments (USA) boards (MATDAQ; H. P. C. Robinson).

#### Logarithmically Stepped Naturalistic Stimulation

In the histamine rescue experiments, the light stimulus was delivered at the center of a R1–R6 photoreceptor’s receptive field, using a Cardan arm system. But in this case, the stimulus presented a sequential light intensity time series mix, delivered by two identical high-performance “white” LEDs (each with a blue–green–red chip-set; dual-channel Cairn OptoLED, UK). Their light outputs were collected by liquid light guides and fused together (to a single end) by a T-connector (Friederich et al., 2009). The LED’s outputs could be attenuated by separate neutral density filter sets. The measured linear light output was taken as the input to the photoreceptors. Its light modulation (stimulus pattern) was selected from the van Hateren’s natural-stimulus-collection (van Hateren, 1997), played back at 2 kHz and measured by a photo diode circuit. Voltage responses (output) and light stimuli (input) were low-pass filtered with a cut-off at 1 kHz before sampling with 2 kHz, and stored for off-line analysis. By driving the two LEDs sequentially through the predetermined neutral density filters, we could change the light level of the stimulus pattern in logarithmic steps rapidly (<0.1 ms). This provided with 0, 1, 2, 3, and 4 log intensity units attenuation, and thus five different light levels (BG0-BG4); BG0 = bright; BG1 = mid; BG3 = dim. Twenty second stimulation at each light level consisted of 10 repetitions of a 2 s naturalistic light intensity time series pattern.

#### Current-Clamp

Electric membrane properties of dark-adapted photoreceptors were investigated by injecting current steps of ±0.04, ±0.13, ±0.21, and ±0.3 nA. Membrane input resistance, *R*_m_, was calculated by the most hyperpolarized voltage (*U*) evoked by a -0.04 nA current step (*I*) according to Ohm’s law:

$$R_{m}=\frac{U}{I}$$

As shown in previous publications (Vähäsöyrinki et al., 2006; Zheng et al., 2006; Abou Tayoun et al., 2011), outcomes of this measurement vary depending on the type of electrode used, room temperature, the experimentalist and other unaccounted factors. To ensure fair comparisons between wild-type and *hdc*^*JK*910^ recordings, we carried out all of these experiments within a week while alternating between the two genotypes and using similar borosilicate electrodes.

### Whole-Cell Recordings

Dissociated ommatidia were prepared from recently eclosed adult flies of either sex and transferred to a recording chamber on an inverted Nikon Diaphot microscope (Hardie et al., 2002). The control bath solution contained the following (in mM): 120 NaCl, 5 KCl, 10 N-Tris-(hydroxymethyl)-methyl-2-aminoethanesulfonic acid (TES), 4 MgCl_2_, 1.5 CaCl_2_, 25 proline, and 5 alanine. Osmolarity was adjusted to ∼283 mOsm. The standard intracellular solution used in the recording pipette was composed of the following (in mM): 140 K^+^ gluconate, 10 TES, 4 Mg^2+^ ATP, 2 MgCl_2_, 1 NAD, and 0.4 Na^+^ GTP. Data were recorded at 20 ± 1°C with an Axopatch 200 amplifier and analyzed with pClamp 9 or 10 software (Molecular Devices). Cells were stimulated by a green (540 nm) LED with intensities calibrated in terms of effectively absorbed photons by counting quantum bumps at low intensities in wild-type flies (Henderson et al., 2000; Hardie et al., 2002). Voltage-gated K^+^-conductances were recorded as explained previously (Hardie, 1991a).

### Electroretinograms

Electroretinograms were recorded from intact flies as previously described (e.g., Satoh et al., 2010). Briefly, female 1–2 weeks old flies were fixed into truncated plastic Gilson pipette tips, using low melting point wax, and stimulated by 1 s light pulses from a red (640 nm) LED with the brightest effective intensity, estimated to be ∼6 × 10^6^ effective photons/photoreceptor/s. Both recording and reference electrodes were filled with fly ringer (in mM): 120 NaCl, 5 KCl, 1.5 CaCl_2_, 4 MgCl_2_, 20 proline, and 5 alanine. The recording electrode was inserted into the retina by piercing the cornea and the indifferent electrode into the head capsule near the ocelli. Recorded signals were low-pass filtered at 200 Hz and amplified via a Neurolog DC amplifier (Digitimer, UK).

Electroretinogram potentials recorded from wild-type *Drosophila* retinae comprise two main components: a maintained background and transients coinciding with changes in light stimuli (Heisenberg, 1971). The maintained background potential (or slow component) is attributed to photoreceptor output and has the inverse waveform of photoreceptors’ intracellular voltage responses, while on- and off-transients originate from the postsynaptic cells in the lamina (Coombe, 1986).

### Electron Micrographs

#### Fixation

Flies were cold anesthetized on ice and transferred to a drop of pre-fixative [modified Karnovsky’s fixative: 2.5% glutaraldehyde, 2.5% paraformaldehyde in 0.1 M sodium cacodylate buffered to pH 7.3 – as per (Shaw et al., 1989)] on a transparent agar dissection dish. Dissection was performed using a shard of a razor blade (Feather S). Flies were restrained on their backs with insect pins through their lower abdomen and distal proboscis. Their heads were severed, proboscis excised, and halved. Left half half-heads were collected in fresh pre-fixative and kept for 2 h at room temperature under normal lighting conditions.

After pre-fixation, the half-heads were washed (2 × 15 min) in 0.1 M Cacodylate buffer, and then transferred to a 1 h post-fixative step, comprising Veronal Acetate buffer and 2% Osmium Tetroxide in the fridge (4°C). They were moved back to room temperature for a 9 min wash (1:1 Veronal Acetate and double-distilled H_2_O mixture), and serially dehydrated in multi-well plates with subsequent 9 min washes in 50, 70, 80, 90, 95, and 2 × 100% ethanol.

Post-dehydration, the half-heads were transferred to small glass vials for infiltration. They were covered in Propylene Oxide (PPO) for 2 × 9 min, transferred into a 1:1 PPO:Epoxy resin mixture (Poly/Bed^®^ 812) and left overnight. The following morning, the half-heads were placed in freshly made pure resin for 4 h, and placed in fresh resin for a further 72 h at 60°C in the oven. Fixation protocol was kindly provided by Professor Ian Meinertzhagen at Dalhousie University, Halifax, Nova Scotia.

#### Sectioning and Staining

Embedded half-heads were first sectioned (at 0.5 μm thickness) using a glass knife, mounted in an ultramicrotome (Reichert-Jung Ultracut E, Germany). Samples were collected on glass slides, stained using Toluidine Blue and observed under a light microscope. This process was repeated and the cutting angle was continuously optimized until the correct orientation and sample depth was achieved; stopping when approximately 40 ommatidia were discernible. The block was then trimmed and shaped for ultra-thin sectioning. The trimming is necessary to reduce cutting pressure on the sample-block and resulting sections, thus helping to prevent “chattering” and compression artifacts.

Ultra-thin sections (85 nm thickness) were cut using a diamond cutting knife (DiATOME Ultra 45°, USA), mounted and controlled using the ultramicrotome. The knife edge was first cleaned using a polystyrol rod to ensure integrity of the sample-blocks. The cutting angles were aligned and the automatic approach- and return-speeds set on the microtome. Sectioning was automatic and samples were collected in the knife water boat.

Sections were transferred to Formvar-coated mesh-grids and stained for imaging: 25 min in Uranyl Acetate; a double-distilled H_2_O wash; 5 min in Reynolds’ Lead Citrate (Reynolds, 1963); and a final double-distilled H_2_O wash.

### Flight Simulator Experiments

We used 3–7 days old *hdc*^*JK*910^ and WT female flies. The flies were tethered in a classic torque meter (Tang and Guo, 2001) with heads fixed, and lowered by a manipulator into the center of a black–white cylinder (spectral full-width: 380–900 nm). A flying fly saw a continuous panoramic scene (360°) of multiple vertical stripes. After viewing the still scene for 1 s, it was spun counterclockwise by a linear stepping motor for 2 s, stopped for 2 s before rotating clockwise for 2 s, and stopped again for 1 s. This 8 s stimulus was repeated 10 times and each trial, together with the fly’s yaw torque responses, was sampled at 1 kHz (Wardill et al., 2012). Flies followed the scene rotations, generating yaw torque responses (optomotor responses to right and left), the strength of which reflects the strength of their motion perception. Stimulus parameters for the moving stripe scenes were as follows: azimuth ± 360°, elevation ± 45°, wavelength 14°, and contrast 1.0, as seen by the fly. The velocity of the scene rotations was 45°/s.

### Data Analysis

The signal was the average of consecutive 1,000 ms long voltage responses to a repeated light intensity time series, selected from the van Hateren naturalistic stimulus library (van Hateren, 1997), and its power spectrum was calculated using Matlab’s Fast Fourier Transform (FFT) algorithm. First 10–20 responses were omitted because of their adaptive trends, and only approximately steady-state adapted responses were analyzed.

The noise was the difference between individual responses and the signal, and its power spectra were calculated from the corresponding traces (Juusola et al., 1994). To eliminate sample-size bias, the same amount of responses (*n* = 60 traces) was used for analyzing each wild-type or *hdc*^*JK*910^ recording to the repeated stimulus. Thus, 60 trials gave one signal trace and 60 noise traces. Both signal and noise data were chunked into 50% overlapping stretches and windowed with a Blackman-Harris-term window, each giving three 500-point-long samples. This gave 180 spectral samples for the noise and three spectral samples for the signal, which were averaged, respectively, to improve the estimates.

#### Triple Extrapolation Method

We used triple extrapolation method (Juusola and de Polavieja, 2003) to estimate the rate of information transfer of steady-state-adapted photoreceptor voltage responses to naturalistic stimulation. This method, unlike signal-to-noise ratio analysis, requires no assumptions about the signal and noise distributions or their additivity (Juusola and de Polavieja, 2003). Voltage responses were digitized by sectioning them into time intervals, *T*, that were subdivided into smaller intervals *t* = 1 ms. (Only dim luminance data was down-sampled to 125 Hz, giving *t* = 8 ms, which better represented their slow dynamics). In the final step, the estimates for the entropy rate, *R*_S_, and noise entropy rate, *R*_N_, were then extrapolated from the values of the experimentally obtained entropies to their successive limits, as in (Juusola and de Polavieja, 2003):

$$R=R_{s}-R_{N}=\underset{T\to \infty }{\lim}\frac{1}{T}\underset{\partial \to \infty }{\lim}\underset{size\to \infty }{\lim}\left(H_{S}^{T,\partial ,size}-H_{N}^{T,\partial ,size}\right)\,$$

where *T* is the length of the “words,” *v* the number of voltage levels (in digitized amplitude resolution) and the size of the data file. The difference between the entropy and noise entropy rates is the rate of information transfer, *R* (Shannon, 1948). See (Juusola and de Polavieja, 2003) for further details.

#### Shannon Information Transfer Rate

To cross-check the triple extrapolation method results, we further estimated information transfer rate, *R*, based on signal-to-noise ratio of photoreceptor responses by using Shannon’s formula:

$$R=\int_{0}^{\infty }\left(\log_{2}\left[SNR(f)+\right]\right)df$$

where *SNR*(*f*) is the signal-to-noise ratio computed for each frequency.

Since a data sampling rate of 1 kHz was used for every naturalistic stimulation experiment, this estimation did not integrate information rate for frequencies from 0 to infinity, but from 2 Hz to 500 Hz instead. However, the limited bandwidth would not considerably affect estimation results because high frequency components have *SNR* <<1 and therefore contain mostly noise.

Finite data can be used to estimate information transfer rate using the Shannon method with the following assumptions: (i) input stimulus is Gaussian, (ii) response is linear and (iii) noise is Gaussian and additive (Shannon, 1948). Thus, estimation accuracy of this method could be affected as these assumptions were not satisfied in photoreceptor responses to naturalistic stimuli (van Hateren and Snippe, 2001; Juusola and de Polavieja, 2003).

Although the triple extrapolation method is not based on assumptions of response and noise statistics, errors could occur in its triple extrapolation to the infinite limit of three finite parameters: data length, time interval, and digitized voltage level.

Nevertheless, both methods, each of which is based on different principles and has different limitations, produced similar estimates and consistent relative comparisons (**Figures 7G** and **8**). Notice also that these estimates were obtained for the recordings at 19 ± 1°C. This temperature was chosen to be akin to *ex vivo* whole-cell recordings for direct comparisons. But as warming improves photoreceptors’ encoding performance (Juusola and Hardie, 2001b), their information transfer rate estimates are higher at higher temperatures; e.g., >400 bits/s for comparable bright naturalistic stimuli at 25°C (Song and Juusola, 2014).

Relative variation, *RV*, was used to approximate the extent of cell-to-cell variations. For measurements or parameters computed from responses of photoreceptors belonging to each group (the wild-type or *hdc*^*JK*910^), relative variation is calculated as:

$$RV=\frac{S\,\tan dard\,\;deviation}{mean}$$

#### Probability Density Functions (PDFs)

Probability density functions (PDFs) were calculated for the 1st, 2nd, and 15th s of photoreceptor responses to Bright NS. Initially, the mean of each 1-s-long response is removed. Then, histograms of their voltage outputs were created by using 2 mV bin size (resolution). Finally, because each response has 1,000 data point, PDFs were calculated by dividing the *y*-axis of histograms by 1,000.

### Statistics

Test responses were compared with their controls by performing *t*-tests. One-tailed tests were used for testing the hypothesis that something was smaller or larger than, while two-tailed tests evaluated the difference in the compared datasets. Welch’s *t*-test was used to accommodate groups with different variances for the unpaired comparisons. In the figures, asterisks are used to mark the statistical significance: ns indicates *p* > 0.05, ^∗^indicates *p* ≤ 0.05, ^∗∗^indicates *p* ≤ 0.01, and ^∗∗∗^indicates *p* ≤ 0.001.

## Results

### *hdc*^*JK*910^ Mutants are Blind because Their Photoreceptors Lack Neurotransmitter

**FIGURE 2:**
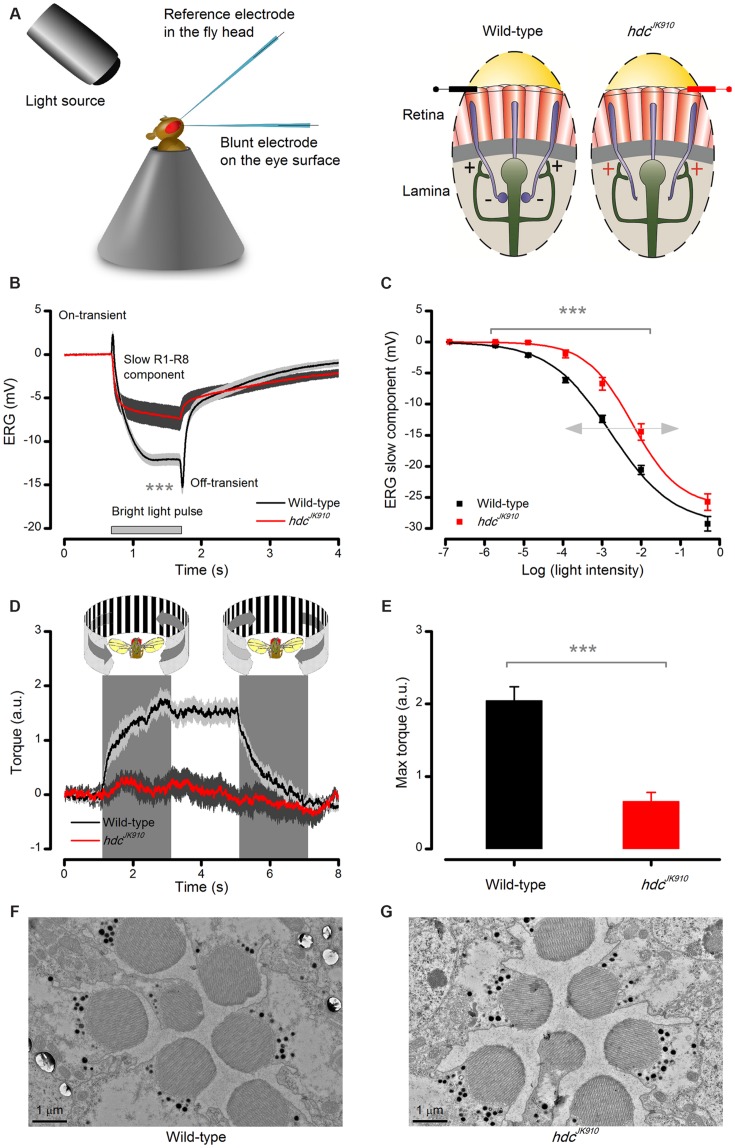
**Lack of photoreceptors’ neurotransmitter histamine makes *hdc*^*JK*910^ mutants blind.** **(A)** Schematic shows how electroretinograms (ERG) were recorded *in vivo* from intact *Drosophila*, restricted inside a conical holder (left). Corneal electrodes (left) were used to pick up electrical signals of light-induced global eye activity, which in the wild-type is dominated by slow photoreceptor and fast lamina interneuron components (on- and off-spikes). In each lamina cartridge, inhibitory (histaminergic) feedforward signals from R1–R6 photoreceptor axons (minus-signs) transmit information from the same small area in the visual space. In return, R1–R6 axons receive excitatory (cholinergic and glutamatergic) feedback signals (plus-signs) from L2/AC and L4 interneurons. *hdc*^*JK*910^ mutants lack the inhibitory feedforward. **(B)** ERGs measured from *hdc*^*JK*910^ and wild-type flies to 1 s long very bright red (640 nm) pulse. Mutant ERGs showed remarkably smaller Photoreceptor Component (_ERG_PC_hdc_ = -7.5 ± 1.3 mV, *_ERG_*PC*_wild_*_-type_ = -14.6 ± 0.8 mV, *p* = 4.594 × 10^-4^, *n*_hdc_ = 7, *n*_wild-type_ = 8 flies) and no on- and off-transients. The large transients in wild-type flies indicate normal neurotransmission from photoreceptors to interneurons. **(C)** *hdc*^*JK*910^ ERG’s photoreceptor component was consistently smaller than that in the wild-type over a broad intensity range (*p* < 3.50 × 10^-4^, one-tailed *t*-test, *n*_hdc_ = 10, *n*_wild-type_ = 10 flies), indicating 10-fold reduced sensitivity. Data fitted by the standard V/logI equation (Hill-equation): $V=V_{\max}\left(\frac{I^{n}}{I^{n}+I_{50}^{n}}\right)$. **(D)** Optomotor responses of tethered flying wild-type and *hdc*^*JK*910^ flies to left (counter-clockwise) and right (clockwise) stripe-field rotations, measured in a classic *Drosophila* flight simulator system. Wild-type flies generate clear and consistent yaw torque responses, intending to follow the field rotations, while the responses of *hdc*^*JK*910^ flies seem haphazard. **(E)** The maximum optomotor responses of *hdc*^*JK*910^ flies are significantly weaker than those of wild-type flies (OR_hdc_ = 0.7 ± 0.1 a.u., OR_wild-type_ = 2.0 ± 0.2 a.u., *p* = 1.56 × 10^-4^, one-tailed *t*-test, *n*_hdc_ = 5, *n*_wild-type_ = 7 flies). **(F)** Characteristic electron micrograph of R1–R7 photoreceptors in wild-type retinae. **(G)** Characteristic electron micrograph of R1–R7 photoreceptors in *hdc*^*JK*910^ retinae. Whilst overall *hdc*^*JK*910^ photoreceptors’ ultrastructure seems normal, the mean R1–R6 rhabdomere cross-sectional area is 28.6% smaller than that of the wildtype (Rhab_hdc_ = 2.05 ± 0.06 μm^2^, Rhab_wild-type_ = 2.87 ± 0.13 μm^2^, *p* = 7.71 × 10^-7^, one-tailed *t*-test, *n*_hdc_ = 18 cells, *n*_wild-type_ = 18 cells, 3 flies). **(B–E)** Mean ± SEM. All recordings performed at *t* = 19°C.

We first asked whether *hdc*^*JK*910^ mutants could see. Although the lack of histamine synthesis should prevent histaminergic information transfer from *hdc*^*JK*910^ photoreceptors to interneurons (Hardie, 1989; Burg et al., 1993; Melzig et al., 1998), some vision might still be possible if the photoreceptors expressed another neurotransmitter or, because of dynamic or homeostatic changes in the lamina network (Zheng et al., 2006; Abou Tayoun et al., 2011), light information were channeled to interneurons through gap-junctions (Wardill et al., 2012). Therefore, we tested *hdc*^*JK*910^ mutants’ sight both electrophysiologically and behaviorally, covering neurotransmission in the eye and any perception in the brain (**Figure 2**).

The eyes’ global electrical responses (ERGs; **Figure 2A**, left) to repeated bright 1 s light pulses were measured with an electrode inserted into the distal retina, which integrated extracellular activity of their photoreceptors and interneurons over a large field of view (right). The recordings (**Figure 2B**) confirmed earlier results that *hdc*^*JK*910^ ERGs completely lack the characteristic on- and off-transients of the wild-type responses (Burg et al., 1993; Melzig et al., 1998), indicating a total block of *hdc*^*JK*910^ photoreceptors’ neurotransmission to interneurons. Interestingly, however, their slow hyperpolarizing components were also consistently smaller (*p* < 0.007) than the wild-type counterparts over a 10^5^ light intensity range (**Figure 2C**), suggesting that *hdc*^*JK*910^ photoreceptors were about 10-fold less sensitive, as seen by the rightward shift in their V/logI-function.

In concordance with the ERG data, tethered flying *hdc*^*JK*910^ mutants in a flight simulator system failed to track bright panoramic field rotations (**Figure 2D**). These findings support the former results about *hdc*^*JK*910^ mutants’ unresponsiveness to pattern luminance changes (Melzig et al., 1996). The mutants could be motivated to fly by airflow, but their flight behavior seemed haphazard and uncoupled to optomotor stimuli, resulting in much smaller maximum responses (**Figure 2E**). In striking contrast, the same stimuli invariably evoked strong reflex-like turns (optomotor responses) in wild-type flies (left), highlighting the perceptual potency of this stimulus paradigm.

Structurally, *hdc*^*JK*910^ photoreceptors were wild-type-like. Electron micrographs from wild-type and *hdc*^*JK*910^ retinae (**Figures 2F,G**, respectively) appeared similar, indicating that *hdc*^*JK*910^ photoreceptors have normal ultrastructure although their rhabdomere dimensions were slightly smaller. Hence, collectively, these results imply that whilst *hdc*^*JK*910^ rhabdomeres may sample light information normally, the mutants are effectively blind because neurotransmission from their photoreceptors to lamina interneurons is missing.

### *hdc*^*JK*910^ Photoreceptors’ Operating Range is Compromised

Based on the ERG data, *hdc*^*JK*910^ photoreceptors fail to transmit light changes to lamina interneurons. However, surprisingly, their slow component, which presumably charts photoreceptor output strength (light sensitivity) and is routinely assumed to be independent of feedforward neurotransmission, was dramatically reduced with respect to that of wild-type flies (**Figure 2B**). Hence, we next asked whether this difference in photoreceptor function could in fact reflect re-balancing of feedback loads in the photoreceptor-interneuron circuits (**Figure 1C**). We reasoned that such re-balancing probably involves both fast dynamic and gradual homeostatic (intrinsic and synaptic ion channel expression) regulation, and that the photoreceptor function, therefore, would reflect their joint contributions.

**FIGURE 3:**
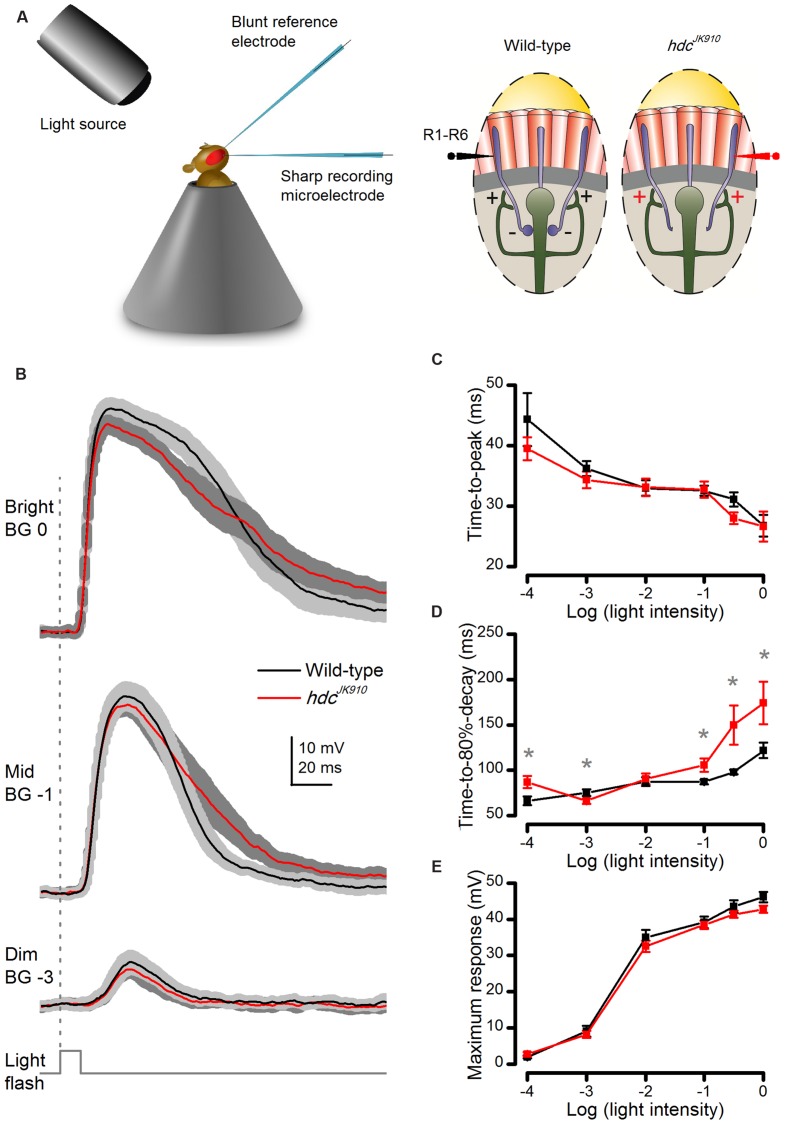
**Dark-adapted *hdc*^*JK*910^ photoreceptors’ voltage responses to brief (10 ms) light pulses rise wild-type-like but decay slower.** **(A)** Schematic shows how *in vivo* microelectrode recordings were performed from R1–R6 photoreceptors. **(B)** Photoreceptors’ voltage responses to Bright, Mid, and Dim 10 ms light pulses. Mutant photoreceptors (red) took longer time than the wild-type (black) to repolarize. **(C)** Response time-to-peak of *hdc*^*JK*910^ and wild-type photoreceptors was typically similar (0.057 < *p* < 0.961, two-tailed *t*-test). **(D)** Average response of *hdc*^*JK*910^ photoreceptors typically lasted longer than that of the wild-type in experiments using brighter flashes. (*p*_Log-4_ = 0.017; *p*_Log-3_ = 0.030; *p*_Log-2_ = 0.350; *p*_Log-1_ = 0.020; *p*_Log-0.5_ = 0.025; *p*_Log0_ = 0.033, one-tailed *t*-test). **(E)** Average response amplitudes of *hdc*^*JK*910^ photoreceptors were wild-type-like (0.071 < *p* < 0.727, two-tailed *t*-test). **(B–E)**: Mean ± SEM; *n*_wild-type_ = 9, *n*_hdc_ = 8. All recordings performed at *t* = 19°C.

We started investigating this question in *dark-adapted* retinae by quantifying whether or how *hdc*^*JK*910^ R1–R6 photoreceptors’ intracellular signaling properties differ from those of wild-type flies (**Figure 3A**). Because R1–R6s are short with high length constants, recordings from their somata can be used to quantify how changes in feedforward and feedback pathways affect their responses (Nikolaev et al., 2009). Their average responses, recorded *in vivo* to brief (10 ms) light pulses of different intensity, are shown in **Figure 3B**. The cells responded with graded depolarizations of similar rise-times over most tested light intensities (**Figure 3C**). But clear differences were seen in *hdc*^*JK*910^ responses to Mid to Bright intensities, which returned to resting potential more slowly than in wild-type photoreceptors (**Figure 3D**), as quantified at 80% of the maxima. Nevertheless, *hdc*^*JK*910^ response amplitudes to brief flashes seemed wild-type-like over the tested light intensity range (**Figure 3E**). Overall, the results suggest that the primary phototransduction mechanisms, which sample photons and integrate the resulting elementary responses (quantum bumps) into macroscopic responses, would be functioning more or less normally in *hdc*^*JK*910^ photoreceptors.

**FIGURE 4:**
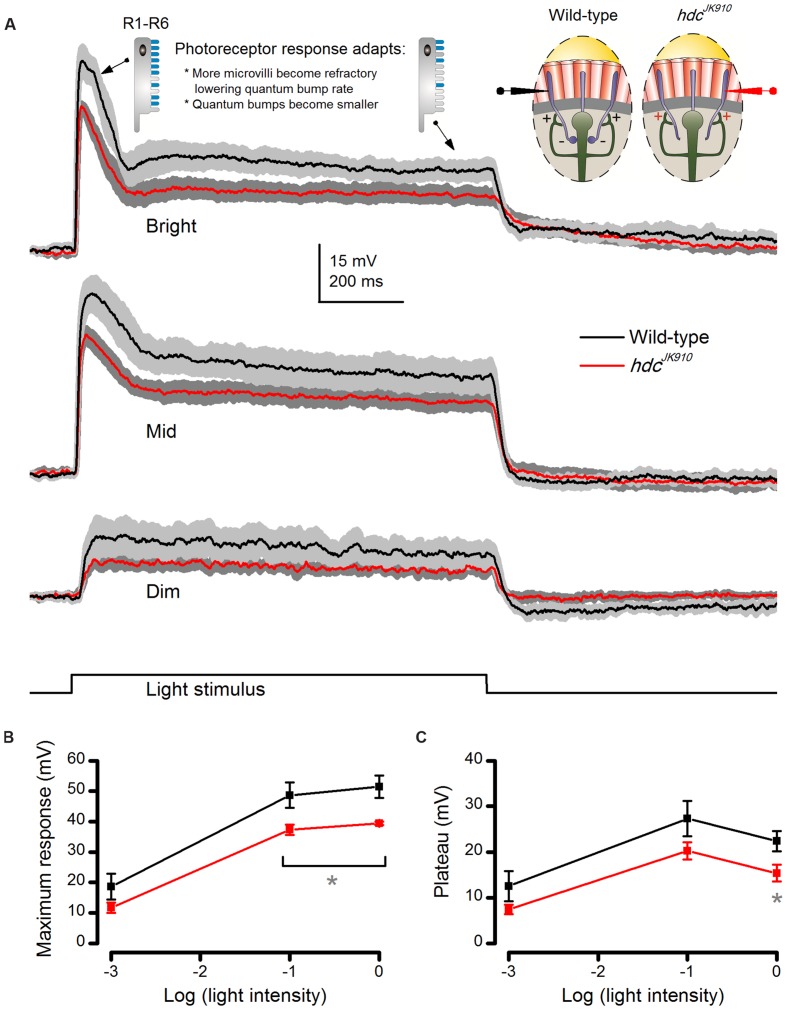
***hdc*^*JK*910^ photoreceptors’ voltage responses to long (1 s) light pulses are smaller than those of the wild-type.** **(A)** On average, *hdc*^*JK*910^ photoreceptors produced smaller responses than the wild-type to long light pulses of all tested intensities, but their responses adapted similarly. The underlying sampling dynamics are described in the main text. **(B)** Their maximum amplitudes differed significantly to Bright and Mid pulses. Dim: Max_wild-type_ = 18.62 ± 4.28 mV, Max_hdc_ = 11.74 ± 1.69 mV, *p* = 0.194. Mid: Max_wild-type_ = 48.60 ± 4.14 mV, Max_hdc_ = 37.23 ± 1.70 mV, *p* = 0.049. Bright: Max_wild-type_ = 51.35 ± 3.64 mV, Max_hdc_ = 39.35 ± 0.57 mV, *p* = 0.029. **(C)** Their plateau voltage differed significantly only in experiments with Bright light pulses. Dim: *P*_wild-type_ = 12.51 ± 3.29 mV, *P*_hdc_ = 7.44 ± 1.04 mV, *p* = 0.204. Mid: *P*_wild-type_ = 27.28 ± 3.86 mV, *P*_hdc_ = 20.24 ± 1.87 mV, *p* = 0.154. Bright: *P*_wild-type_ = 22.36 ± 2.23 mV, *P*_hdc_ = 15.37 ± 1.82 mV, *p* = 0.043. **(A–C)**: *n*_wild-type_ = 5, *n*_hdc_ = 5; Mean ± SEM, two-tailed *t*-test. All recordings performed at *t* = 19°C.

Next, to compare how *progressive light adaptation* affects *hdc*^*JK*910^ and wild-type photoreceptor outputs, we recorded their voltage responses to prolonged (1 s) Dim (-3), Mid (-1), and Bright (0 log unit) light pulses (**Figure 4A**). A fly photoreceptor’s typical response waveform comprises an initial peak and a plateau, which can be partly explained by its refractory photon sampling characteristics (Song et al., 2012). After sufficient dark-adaptation, most of its ∼30,000 microvilli (sampling units) are available to produce quantum bumps to absorbed photons, summation of which constitutes the initial peak. From then on, fewer microvilli can be photon-activated because many are now refractory, a period which lasts 50–300 ms. Therefore, depending on the light pulse intensity and the number of microvilli used in the previous phase, the plateau can be significantly lower than the peak. Accordingly, wild-type photoreceptors showed large initial peaks in responses to Mid and Bright but not to Dim light pulses. Moreover, bump size becomes reduced under brighter stimulation due to Ca^2+^-dependent feedback and reduced EMF as the cell depolarizes (Henderson et al., 2000; Juusola and Hardie, 2001a; Song et al., 2012), contributing more to the smaller plateau during the Bright than the Mid pulse. Crucially, *hdc*^*JK*910^ photoreceptors exhibited wild-type-like waveforms (**Figure 4A**) but with smaller peak (**Figure 4B**) and plateau amplitudes (**Figure 4C**). This, together with the *hdc*^*JK*910^ photoreceptors’ normal ultrastructure (**Figures 2F,G**), suggests that their photon sampling and bump summation would occur normally (or near-normally), but that their overall voltage output (operating range) was compressed by separate mechanisms.

### *hdc*^*JK*910^ Mutation Affects Photoreceptors’ Electrical Properties

**FIGURE 5:**
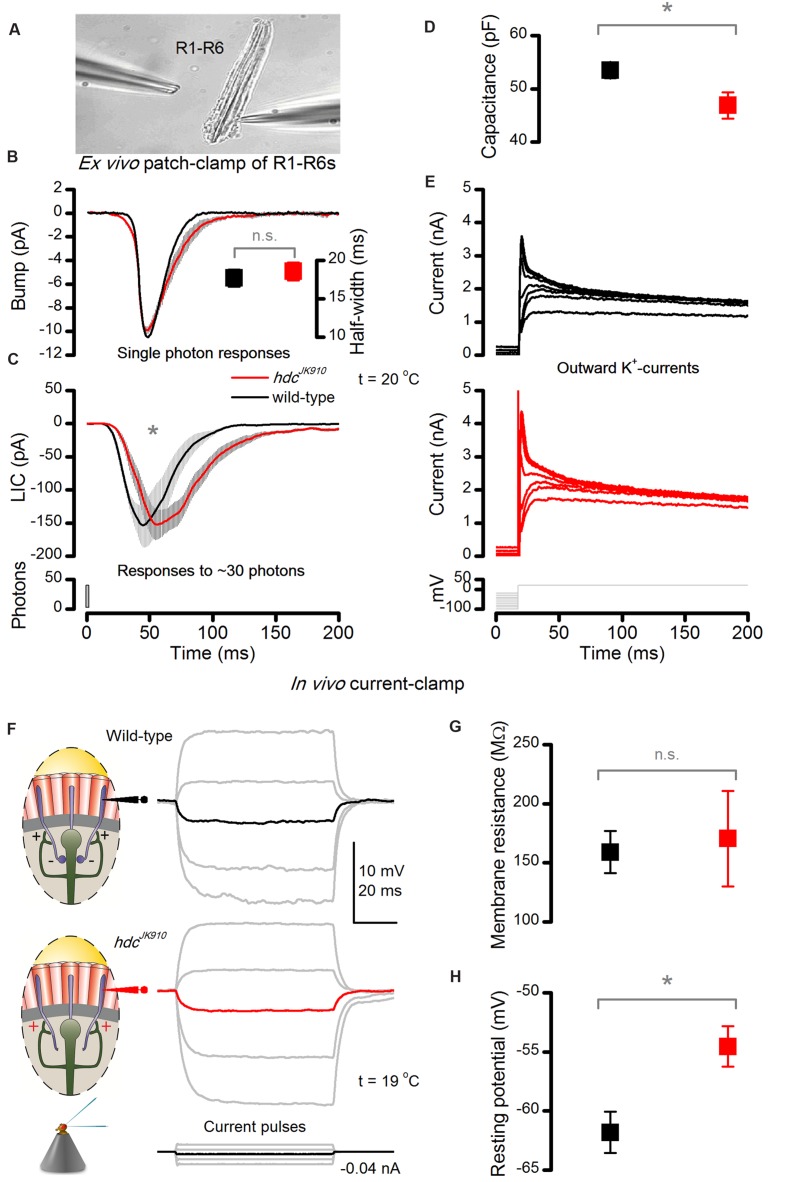
***hdc*^*JK*910^ photoreceptors’ macroscopic light-induced currents (LIC) peak and decay slower *ex vivo* and their *in vivo* dark resting potentials are higher than the wild-type.** **(A)** LICs and voltage-gated K^+^-currents were patch-clamp recorded from *hdc*^*JK*910^ and wild-type R1–R6 photoreceptors in ommatidia, dissociated from freshly eclosed flies. The image is adapted from (Abou Tayoun et al., 2011). **(B)** Characteristic *hdc*^*JK*910^ and wild-type single photon responses and their half-widths (insert). Bumps have a similar time course and mean sizes (bump_hdc_ = 13.9 ± 0.8 pA, bump_wild-type_ = 16.2 ± 1.4 pA; *p* = 0.158, two-tailed *t*-test, *n*_hdc_ = 8, *n*_wild-type_ = 5). Quantum efficiency was not significantly different (QE_hdc_ = 0.62 ± 0.04, QE_wild-type_ = 0.64 ± 0.02; *p* = 0.805, two-tailed *t*-test, *n*_hdc_ = 8, *n*_wild-type_ = 5). **(C)** Macroscopic *hdc*^*JK*910^ LICs showed slower than wild-type dynamics. (time-to-peak_hdc_ = 60.4 ± 9.7 ms, time-to-peak_wild-type_ = 46.1 ± 8.1 ms; *p* = 0.008, one-tailed *t*-test, *n*_hdc_ = 5, *n*_wild-type_ = 8). **(D)** Interestingly, the *hdc*^*JK*910^ membrane capacitance was lower than the wild-type (*C*_hdc_ = 46.9 ± 5.5 pF, *C*_wild-type_ = 53.5 ± 4.2 pF; *p* = 0.016, one-tailed *t*-test, *n*_hdc_ = 5, *n*_wild-type_ = 8). Mean leak currents did not differ significantly (leak_hdc_ = -8.2 ± 10.2 pA, leak_wild-type_ = -10.1 ± 9.0 pA; *p* = 0.720, two-tailed *t*-test, *n*_hdc_ = 5, *n*_wild-type_ = 8). **(E)** Voltage-gated K^+^-currents of *hdc*^*JK*910^ and wild-type R1–R6 photoreceptors show similar amplitudes and dynamics; these representative traces are well within the natural variability. **(F)** Voltage response of dark-adapted wild-type and *hdc*^*JK*910^ photoreceptors, recorded *in vivo* to intracellularly injected current pulses. **(G)** Membrane resistance of *hdc*^*JK*910^ photoreceptors are wild-type-like (_m_*R*_hdc_ = 170.5 ± 40.4 MΩ, _m_*R*_wild-type_ = 159 ± 17.8 MΩ; *p* = 0.807, two-tailed *t*-test, *n*_hdc_ = 4, *n*_wild-type_ = 4). **(H)** In darkness, the resting potential of *hdc*^*JK*910^ photoreceptors is significantly higher than in the wild-type (*V*_hdc_ = -54.5 ± 1.7 mV, *V*_wild-type_ = -61.8 ± 1.74 mV; *p* = 0.011, one-tailed *t*-test, *n*_hdc_ = 4, *n*_wild-type_ = 5). **(D–H)** Mean ± SEM. All recordings performed at *t* = 19°C.

A photoreceptor’s voltage response is the outcome of a complex convolution of three components: light-induced currents (LICs), light-insensitive membrane conductances and synaptic feedback from interneurons (Zheng et al., 2006; Abou Tayoun et al., 2011). Therefore, to explain the differences in response characteristics of the mutant and wild-type photoreceptors, it is essential to compare their component properties in isolation (**Figure 5**).

Phototransduction dynamics and membrane properties of *hdc*^*JK*910^ R1–R6 photoreceptors were examined *ex vivo* (**Figure 5A**) by whole-cell patch-clamping photoreceptors in dissociated ommatidia that were cut off from the lamina synaptic network (Hardie, 1991b). The recordings, which were thus free of any interneuron feedback, revealed some interesting differences to wild-type controls. Firstly, even though *hdc*^*JK*910^ photoreceptors’ single-photon-responses (current bumps) had similar amplitudes and dynamics (**Figure 5B**), their macroscopic LICs to 1 ms light flashes rose and decayed more slowly than those of wild-type photoreceptors, with the response decay being considerably more decelerated (**Figure 5C**). Nevertheless, the cells’ quantum efficiency appeared similar, indicating that their microvilli sampled photons with similar success. Secondly, the membrane capacitance (*C*_m_) of *hdc*^*JK*910^ photoreceptors seemed slightly lower than wild-type (**Figure 5D**), which is consistent with their slightly smaller rhabdomere membrane areas (**Figures 2F,G**), but their somatic K^+^-conductances were similar to those of wild-type photoreceptors (**Figure 5E**).

Of these findings, the slower LIC dynamics in *hdc*^*JK*910^ (**Figure 5C**) contrast with our *in vivo* observations the most (cf. **Figure 3B**); as in the intact retina, the mutant and wild-type impulse responses showed largely uniform rise-time dynamics. An explanation for this difference could be that homeostatic network regulation partially restores the dynamic coding of light intensities *in vivo*. However, because *hdc*^*JK*910^ lamina interneurons do not receive synaptic information from photoreceptors, their feedback signal is expected to be independent of light intensity. Thus, two questions arise here: how does such tonic interneuron feedback affect photoreceptors’ *in vivo* membrane conductances? And, how do light-sensitive and light-insensitive conductances and synaptic feedback jointly shape *hdc*^*JK*910^ photoreceptor output?

To discover the impact of interneuron feedback on the membrane when feedforward synaptic pathways are blocked and without the LIC interference, we investigated *in vivo* membrane properties of dark-adapted photoreceptors with single-electrode current-clamp technique. We discovered that voltage responses to injected current steps were indistinguishable in wild-type and *hdc*^*JK*910^ mutant photoreceptor somata (**Figure 5F**). Moreover, their membrane input resistances, *R*_m_, which were calculated from hyperpolarizing responses to a small negative current step (-0.04 nA) to minimize activation of voltage-gated K^+^ channels (Hardie, 1991a), showed similar ranges (**Figure 5G**).

But perhaps most interestingly, the resting potentials of mutant photoreceptors, which had stable responses for 20 min or longer, were regularly ∼6 mV more depolarized than their wild-type counterparts in darkness (**Figure 5H**). This implies that lack of inhibitory (histaminergic) inputs to *hdc*^*JK*910^ LMCs and ACs depolarize these cells tonically – both in darkness and in light (Laughlin et al., 1987; Uusitalo et al., 1995a), which in turn increases their tonic excitatory load to R1–R6s.This finding and explanation concur with *shibire*^*TS*1^ mutant recordings (Zheng et al., 2006), which showed that silencing all synaptic transmission, including the LMC and AC feedback to R1–R6s, hyperpolarize photoreceptors. Thus, with the earlier *shibire*^*TS*1^ data, *hdc*^*JK*910^ R1–R6 depolarization here indicates increased excitatory interneuron feedback to them, and opposes the alternative hypothesis of reduced inhibitory (hyperpolarizing) feedback.

Taken together, these results suggest that *hdc*^*JK*910^ photoreceptors receive *in vivo* more depolarizing conductances from interneuron feedback(s) than wild-type cells; but further intrinsic compensatory mechanisms, such as possible down-regulation of leak-channels (Vähäsöyrinki et al., 2006), may restore their membrane resistance to a wild-type level, or the real differences might be masked by a shunt introduced by the electrode penetration. Thereby, it seems plausible that the disrupted interneuron feedback, caused by blocking feedforward synaptic transmissions, would alter the dynamic equilibrium of ion channels in the photoreceptor cell membrane. The observed reduction in *hdc*^*JK*910^ photoreceptors’ membrane capacitance, which agrees with their slightly smaller rhabdomeres (**Figures 2F,G**), would further lower their membrane time constant (*t*_m_ = *R*_m_ × *C*_m_), accelerating response conduction. Naturally, all these changes would impact signal conduction most at the level of axon terminals, which receive the interneuron feedback. In general, this scenario seems consistent with the photoreceptors’ response kinetics to 10 ms light pulses, as recorded in the retina (**Figure 3C**). Convolution of *hdc*^*JK*910^ LICs, which would still recover slower (despite speedier axonal conduction), with (near) normal somatic membrane properties should yield responses with wild-type-like rising and slower decaying phases.

### *hdc*^*JK*910^ Photoreceptors Adapt Wild-Type-Like to Repetitive Dark-to-Light Stimuli

Modulation within the lamina network can have important contributions to dynamic and homeostatic regulation of photoreceptor output during *naturalistic light stimulation*, as shown by recent findings (Abou Tayoun et al., 2011). Despite having normal *ex vivo* properties, photoreceptors of *dSK*^-^ mutants with altered lamina network, due to missing Ca^2+^-activated K^+^-channels, and thus abnormal feedback signals, showed dark-to-light adapting trends *in vivo* that differed markedly from the wild-type.

**FIGURE 6:**
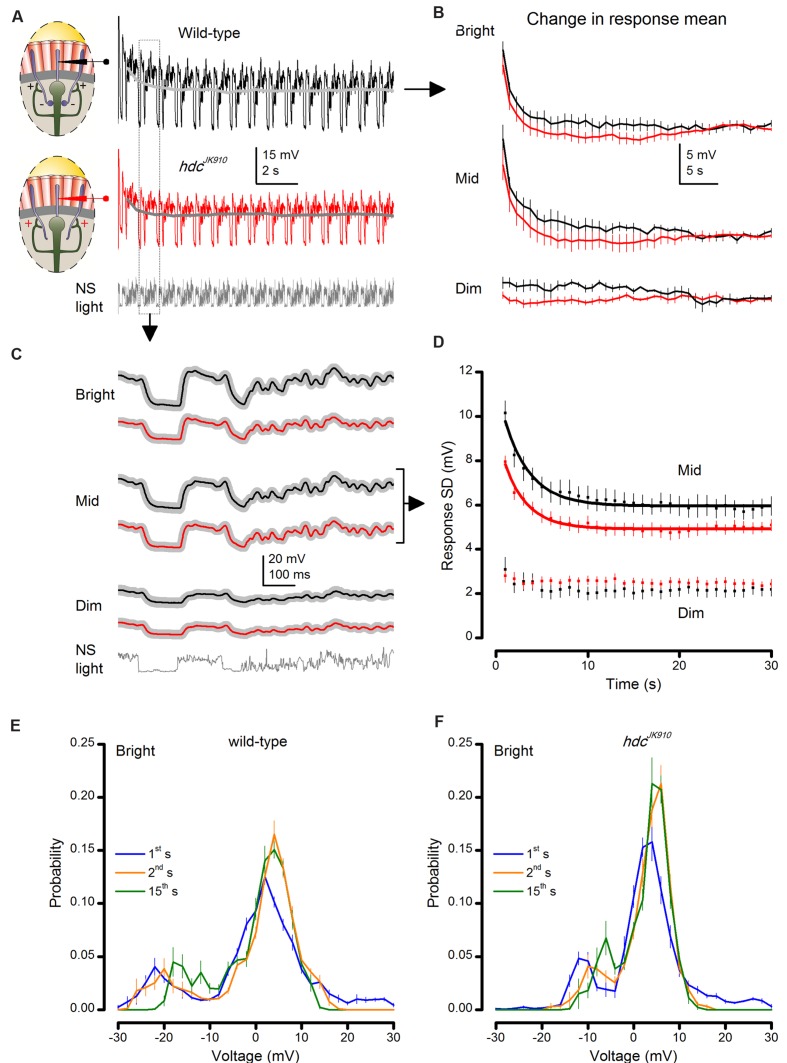
***hdc*^*JK*910^ and wild-type photoreceptor outputs adapt to naturalistic stimulation with similar time courses.** **(A)** Voltage responses of wild-type and *hdc*^*JK*910^ photoreceptors to repeated 1 s long bright naturalistic light intensity time series (NS). **(B)** Change in the mean of 1 s long response over 40 s of stimulation. Differences between mean wild-type and *hdc*^*JK*910^ responses were not statistically significant (*p* = 0.378 ± 0.035, 0.019 ≤*p* ≤ 0.949, across 60 time-bins, two-tailed *t*-test). **(C)** Average waveforms of steady-state adapted 1 s long voltage responses. **(D)** Change in response modulation (Standard Deviation of each 1 s long response) over 30 s of stimulation. Data for Mid light intensities are fitted with exponential curves: *T*_wild-type_ = 2.83 ± 0.06 s, *T*_hdc_ = 2.49 ± 0.07 s. **(E)** Probability Density Functions (PDFs) of wild-type photoreceptor output in the 1st, 2nd, and 15th s of Bright naturalistic stimulation. **(F)** PDFs of *hdc*^*JK*910^ photoreceptor output in the 1st, 2nd, and 15th s of Bright naturalistic stimulation. **(B–F)** Mean ± SEM, *n*_wild-type_ = 7, *n*_hdc_ = 8. All recordings performed at *t* = 19°C.

We, therefore, analyzed how concerted actions of the tonic interneuron feedback, *naturalistic* LICs and intrinsic compensations shape photoreceptor output over time. *hdc*^*JK*910^ and wild-type R1–R6 outputs were recorded to repeated naturalistic stimulation at three different brightness levels (**Figure 6A**).

We found that *hdc*^*JK*910^ photoreceptors’ dark-to-light adapting trends were remarkably similar, in striking contrast to *dSK*^-^ mutant data (Abou Tayoun et al., 2011). Their similar time-course is evident in **Figure 6B**, which depicts the changes in mean of each 1 s long response (or response mean) to stimulus repetition. The corresponding response means relaxed to less depolarized steady levels in ∼15–20 s during Bright or Mid stimulation, but remained relatively unchanged during Dim stimulation. Although the average *hdc*^*JK*910^ response mean appeared to settle faster than that of wild-type flies at each illumination level, this difference was not significant.

Likewise, the analyses revealed that their corresponding waveform (**Figure 6C**) or output modulation, measured by the standard deviation, changed comparably (**Figure 6D**). Responses of *hdc*^*JK*910^ photoreceptors to Bright (not shown) stimulation were consistently smaller than in wild-type photoreceptors but adapted in parallel exponential trends, reaching steady-state ranges equally fast.

Moreover, *hdc*^*JK*910^ photoreceptors’ PDF, which indicate how their output ranges were utilized to represent light intensity modulations, adapted over time in a similar manner to wild-type photoreceptors (**Figure 6E**). Consistent with the previous recordings (Zheng et al., 2009), none of the examined R1–R6 outputs exhibited flattening or widening PDFs, which instead are often seen in the postsynaptic wild-type LMC output.

### *hdc*^*JK*910^ Photoreceptor Output to Bright Stimuli Carries Less Signal Power than Wild-Type

**FIGURE 7:**
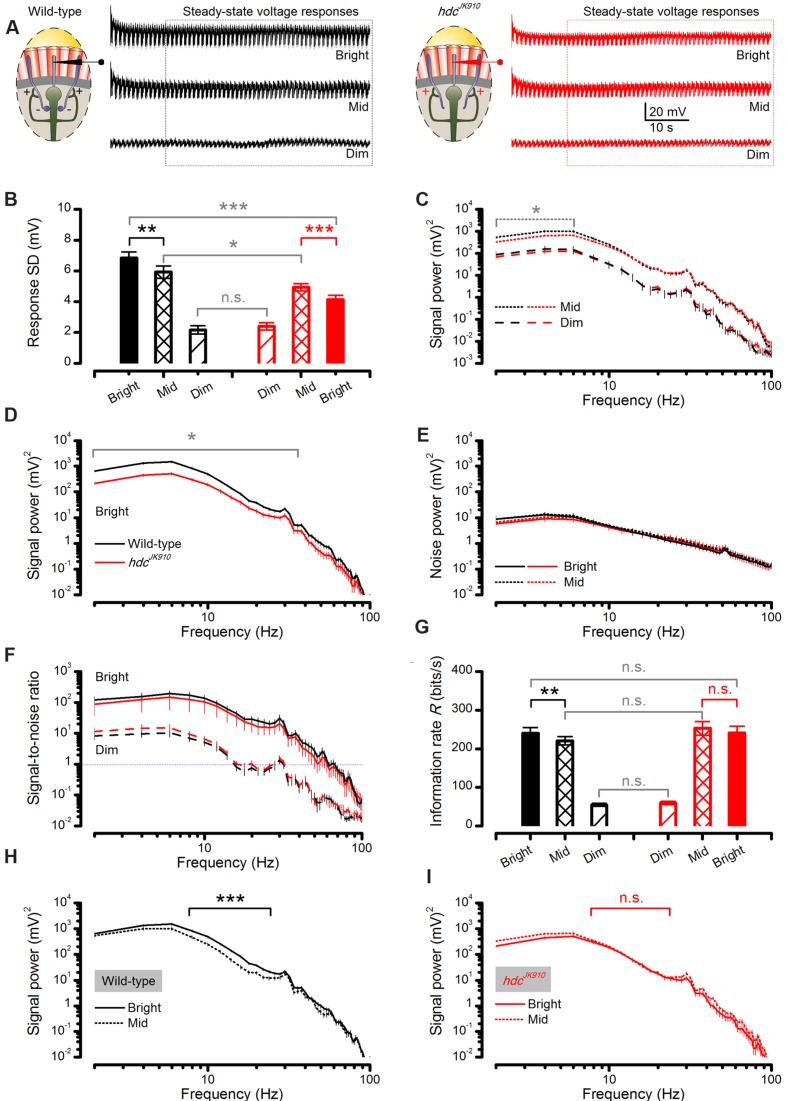
**Steady-state-adapted *hdc*^*JK*910^ photoreceptor output to Mid and Bright naturalistic stimulation show less modulation but their information transfer is remarkably similar to the wild-type.** **(A)** Mean wild-type (black) and *hdc*^*JK*910^ (red) R1–R6 photoreceptors’ voltage response to a repeated 1 s long naturalistic light intensity time series pattern of different brightness. **(B)** In Bright and Mid, *hdc*^*JK*910^ R1–R6 output had significantly less modulation than the wild-type (Bright: *SD*_wild-type_ = 6.85 ± 0.37 mV, *SD*_hdc_ = 4.13 ± 0.27 mV, *p* = 4.427 × 10^-5^, one-tailed *t*-test; Mid: *SD*_wild-type_ = 5.93 ± 0.40 mV, *SD*_hdc_ = 4.92 ± 0.23 mV, *p* = 0.028, one-tailed *t*-test). In Bright, wild-type output modulation was the largest (*p* = 0.007, one-tailed paired *t*-test) while *hdc*^*JK*910^ modulation reduced in respect to Mid (*p* = 2.980 × 10^-4^, one-tailed paired *t*-test). In Dim, *hdc*^*JK*910^ and wild-type outputs showed similar modulation (Dim: *SD*_wild-type_ = 2.16 ± 0.27 mV, *SD*_hdc_ = 2.40 ± 0.23 mV, *p* = 0.520, two-tailed *t*-test). **(C)** In Mid, *hdc*^*JK*910^ signal had less power than the wild-type only in low frequencies (*f* < 6Hz, *p* < 0.029, one-tailed t-test). In Dim, *hdc*^*JK*910^ and wild-type signal power spectra were similar. **(D)** In Bright, *hdc*^*JK*910^ R1–R6 output had less signal power than the wild-type over a broad frequency range (*f* < 34Hz, *p* < 0.023, one-tailed *t*-test). **(E)** Noise power spectra of wild-type and *hdc*^*JK*910^ photoreceptor outputs were similar to all three tested brightness levels. Data in Dim condition is not shown for clarity. **(F)** Signal-to-noise ratios of wild-type and *hdc*^*JK*910^ photoreceptor outputs were remarkably similar over the three tested brightness levels. Data for Mid stimulation are not shown for clarity. **(G)** Information transfer rate estimates of wild-type and mutant photoreceptor outputs were remarkably similar (Dim: *R*_wild-type_ = 54.73 ± 3.67, *R*_hdc_ = 59.53 ± 4.23; Mid: *R*_wild-type_ = 220.44 ± 10.75, *R*_hdc_ = 252.92 ± 17.86; Bright: *R*_wild-type_ = 240.40 ± 14.62, *R*_hdc_ = 240.64 ± 17.41, all in bits/second, *p* > 0.05, two-tailed *t*-test). Wild-type photoreceptors showed higher information transfer rates in Bright stimulation than in Mid (*p* = 0.004, one-tailed paired *t*-test) while *hdc*^*JK*910^ photoreceptors encoded similar information rates in Mid and Bright (*p* = 0.138, two-tailed paired *t*-test). **(H)** For 8–24 Hz frequency range, wild-type photoreceptor outputs to Bright naturalistic stimulation carried significantly more power than to Mid intensities (*p* < 0.001, paired one-tailed *t*-test). **(I)** *hdc*^*JK*910^ photoreceptor outputs to Bright and Mid naturalistic stimulations had similar power spectra (*p* > 0.05, paired two-tailed *t*-test). **(A–I)**: Mean ± SEM, *n*_wild-type_ = 7, *n*_hdc_ = 8. All recordings performed at *t* = 19°C.

We next asked whether or how the *hdc*^*JK*910^ mutation affects the photoreceptors’ ability to sample light information and the way this information is represented in their responses. To obtain consistent and systematic estimates of *hdc*^*JK*910^ and wild-type photoreceptors’ encoding performance, the first 10–20 1 s long responses with adapting trends were discarded, and we analyzed their steady-state adapted responses to the repeated naturalistic stimuli (**Figure 7A**).

We found that their responses to Dim stimulation were of similar size, but *hdc*^*JK*910^ photoreceptor output range (right, red) contracted, while the wild-type range (left, black) expanded, from Mid to Bright stimulation (**Figure 7B**), as partly suggested by their adapting trends (**Figures 6D–F**). Accordingly, when analyzed across the whole populations, *hdc*^*JK*910^ photoreceptors’ mean responses (signals) had wild-type-like power spectra to Dim stimulation but carried significantly less power to Mid, at low frequencies, (**Figure 7C**) and especially to Bright, over a broad frequency range (**Figure 7D**). Here, these large differences are plotted in a logarithmic scale to reveal the full spread of their frequency components. Interestingly, though, noise power spectra in *hdc*^*JK*910^ recordings, as the difference in frequency components between the signal and the individual responses, were similar to the wild-type noise spectra and remained largely unaffected at the different stimulation intensities (**Figure 7E**). Because the noise power spectrum largely represents the average quantum bump’s frequency composition (Juusola and Hardie, 2001a; Song et al., 2012; Song and Juusola, 2014), wild-type and *hdc*^*JK*910^ bumps (or samples) adapted to a similar size. Therefore, the larger wild-type responses to Bright stimulation (**Figure 7B**) simply comprised more samples. Together, these findings predict that *hdc*^*JK*910^ R1–R6s’ encoding performance would fall short of wild-type performance with brightening illumination.

**FIGURE 8:**
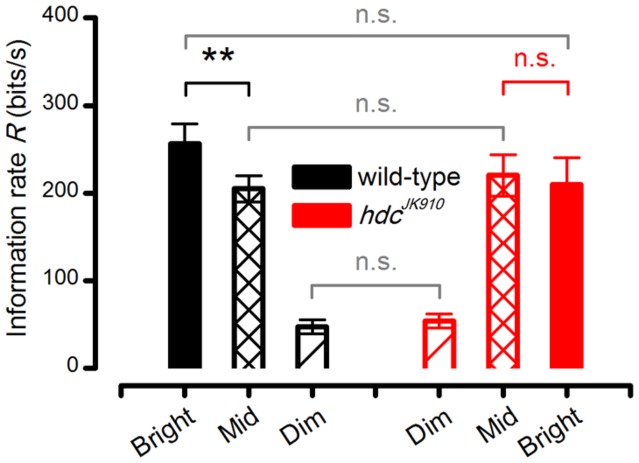
***hdc*^*JK*910^ and wild-type R1–R6 photoreceptors’ Information transfer rates calculated by Shannon’s estimation method.** The cells’ signaling performance to the same naturalistic stimulation was estimated at different intensity levels. Dim: *R*_wild-type_ = 47.00 ± 8.12, *R*_hdc_ = 53.88 ± 7.93; Mid: *R*_wild-type_ = 205.00 ± 14.78, *R*_hdc_ = 220.38 ± 23.71; Bright: *R*_wild-type_ = 256.29 ± 22.98, *R*_hdc_ = 210.25 ± 30.05, all in bits/second. Wild-type photoreceptors showed higher information transfer rates in Bright stimulation than in Mid (*p* = 0.004, paired one-tailed *t*-test). Mean ± SEM, *n*_wild-type_ = 7, *n*_hdc_ = 8. All recordings were performed at *t* = 19°C.

However, when we consider the outputs of individual photoreceptors, *hdc*^*JK*910^ R1–R6s showed remarkably similar signal-to-noise ratios (**Figure 7F**) to those of wild-type at the three intensity levels tested and hence could communicate comparable information rates, *R* (**Figure 7G**). To ensure that these statistically insignificant differences were not errors introduced by the extrapolation method for estimating the information rates (Juusola and de Polavieja, 2003), we also calculated the photoreceptors’ encoding performance using Shannon’s estimation method (Shannon, 1948). In concordance with previous reports (Juusola and de Polavieja, 2003; Song and Juusola, 2014), this produced only slightly different *R* values (**Figure 8** difference 6.3–14.5%), supporting the comparative relations shown in **Figure 7G**.

The apparent contradiction between the population averages and individual responses originates from the large cell-to-cell variations within each group. Average relative variations (computed as SD/mean) of signal, noise and signal-to-noise ratio in wild-type responses (for frequencies ≤ 40 Hz) to Bright naturalistic stimulation were 32, 45, and 70%, respectively. The corresponding values for *hdc*^*JK*910^ responses were 53% (signal power), 78% (noise power) and 141% (signal-to-noise ratio). Evidently, individual differences were intensified by the mathematical relationship of (signal power spectrum)/(noise power spectrum), resulting in a remarkably larger relative variation in signal-to-noise ratio than those in signal and noise power measurements. Therefore, the statistically significant difference in the average signal powers carried by *hdc*^*JK*910^ and wild-type responses was undermined, leading to insignificant differences in their average signal-to-noise ratios and encoding performances.

### *hdc*^*JK*910^ Photoreceptors Reach Maximal Encoding at Lower Intensities than Wild-Type

To further assess how *hdc*^*JK*910^ photoreceptors’ operational range differs from that of wild-type photoreceptors at daylight intensities, we analyzed individual photoreceptors’ responses to Mid and Bright naturalistic stimuli. These paired comparisons reduced the cell-to-cell variation effects, making it easier to quantify common photoreceptor output changes when the stimulation is brightened by one log-unit.

We found that from Mid to Bright, wild-type photoreceptors’ operational range expanded by ∼15% (**Figure 7B**, black bars), increasing signal power significantly at 8–22 Hz frequencies (**Figure 7H**). As noise power stayed effectively unchanged (**Figure 7E**, solid and dotted black lines), information transfer rate estimates of all the tested wild-type photoreceptors (*n* = 7), calculated by triple extrapolation method, improved by ∼10% on average (**Figure 7G**, black bars). A larger *R* value increase (∼25%) was computed by using Shannon’s estimation method (**Figure 8**, black bars). Nonetheless, both estimation methods indicated that this improvement was significant with a similar *p*-value of ∼0.004.

Conversely, all *hdc*^*JK*910^ photoreceptors (*n* = 8) showed the opposite trend with markedly smaller responses. However, their ∼16% response size reduction (**Figure 7B**, red bars) mostly reflected decreased low frequency signal power (**Figure 7I**), which thus carried little information. Hence, their encoding performance did not lower significantly (**Figures 7G** and **8**, red bars). Altogether, *hdc*^*JK*910^ photoreceptors’ information transfer rate estimates showed ∼5% reduction on average, with two out of eight cells showing minor increases.

### *hdc*^*JK*910^ Photoreceptors Lack Phasic Modulation from Interneuron Feedback

The mean of the two independent information rate estimates (**Figures 7G** and **8**) for *hdc*^*JK*910^ photoreceptor output (225.4 ± 21.5 bits/s) during Bright naturalistic stimulation is ∼9% lower than that for the corresponding wild-type output (248.3 ± 11.2 bits/s). This relative performance difference is consistent with the hypothesis that each R1–R6 photoreceptor, within the same lamina cartridge, receives additional information [synaptic quanta (sample) rate changes] from interneuron feedback (Zheng et al., 2006). On balance, interneuron feedback may carry higher information rates than individual photoreceptor outputs because it integrates information from six R1–R6s, which sample light changes from the same small area. Such extra information returning to individual photoreceptors in high signal-to-noise ratio stimulus conditions (i.e., Mid and Bright) is predicted to be in the form of phasic modulation (Zheng et al., 2006).

To test the interneuron feedback hypothesis and its predictions decisively, we devised a new two part experiment.

**FIGURE 9:**
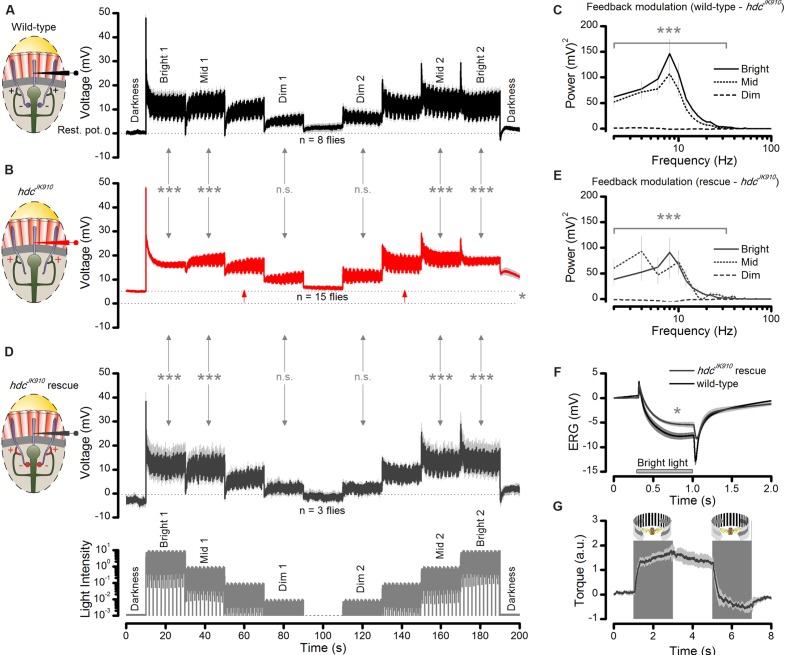
**Interneuron feedback accentuates modulation in R1–R6 photoreceptor output.** **(A)** Voltage responses of wild-type photoreceptors to up- and down-stepped logarithmic naturalistic light changes show strong modulation during Bright, Mid and Dim intensities. Mean ± SEM, *n* = 8 cells. To ease comparison, zero ordinate marks the cell’s average resting potential in darkness. **(B)** *hdc*^*JK*910^ photoreceptors’ responses to the same stimulus exhibit reduced modulation during Bright and Mid intensities; Mean ± SEM shown, *n* = 15 cells. Testing hypotheses that mean wild-type modulation ≠ mean *hdc*^*JK*910^ modulation: Bright 1 (*p* = 1.785 × 10^-8^); Mid 1 (*p* = 3.357 × 10^-7^); Dim 1 (*p* = 0.676); Dim 2 (*p* = 0.292); Mid 2 (*p* = 9.447 × 10^-9^); Bright 2 (*p* = 6.416 × 10^-10^). On average, the cell’s resting potential was 5.3 mV higher than in the wild-type (red arrows between dotted lines, *p* = 0.013, one-tailed *t*-test). **(C)** The difference in the corresponding wild-type (normal feedback) and *hdc*^*JK*910^ (only tonic feedback) response power spectra suggests that the normal interneuron feedback accentuates R1–R6 output, phasically modulating it over 2–44 Hz frequency range during Mid and Bright stimulation. The corresponding wild-type power spectra (feedback modulation) differed from the *hdc*^*JK*910^ power spectra during Bright 1: 1–36 Hz (4.661 × 10^-7^ < *p* < 0.01) and 36–44 Hz (0.019 < *p* < 0.05) and Mid 1 stimuli: 1–18 Hz (1.051 × 10^-6^ < *p* < 0.015). **(D)** Histamine uptake rescues modulation in R1–R6 photoreceptor output in *hdc*^*JK*910^ mutants to wild-type levels; Mean ± SEM shown, *n* = 3 cells. Testing hypotheses that mean *hdc*^*JK*910^ rescue modulation ≠ mean *hdc*^*JK*910^ modulation in Bright 1 (*p* = 9.966 × 10^-7^); Mid 1 (*p* = 1.593 × 10^-4^); Dim 1 (*p* = 0.514); Dim 2 (*p* = 0.693); Mid 2 (*p* = 2.051 × 10^-5^); Bright 2 (*p* = 5.468 × 10^-6^). The rescued cells’ resting potentials differed from the non-rescued cells (*p* = 0.021) but not from the wild-type (*p* = 0.384). **(E)** Histamine-rescue recovers *hdc*^*JK*910^ photoreceptors’ output modulation power spectra to near wild-type levels; cf. (C). The rescued power spectra (with feedback modulation) differ from the *hdc*^*JK*910^ power spectra during Bright 1: 1–34 Hz (9.399 × 10^-7^ < *p* < 0.008) and 36 Hz (*p* = 0.0159) and Mid 1 stimuli: 1–14 Hz (8.425 × 10^-6^ < *p* < 0.005). **(F)** Histamine uptake rescues on- and off-transients in *hdc*^*JK*910^ ERG, indicating that the mutants see. For the given stimulus, the mutants fed on histamine, their ERG’s slow photoreceptor component differs from that of the mutants on normal diet (_ERG_PC_rescue_ = 5.9 ± 1.7 mV, _ERG_PC_hdc_ = 3.6 ± 1.0 mV; mean ± SD, *p* = 0.006, *n*_rescue_ = 4, *n*_hdc_ = 7 flies). However, the rescued photoreceptor component still does not fully match the wild-type ERG (_ERG_PC_wild-type_ = 8.2 ± 1.8 mV; mean ± SD, *p* = 0.034, *n*_wild-type_ = 8 flies); cf. **Figure 2B**. **(G)** Histamine uptake rescues normal optomotor behavior in *hdc*^*JK*910^ mutants, as tested in the *Drosophila* flight simulator system to left and right rotating panoramic stripe patterns; cf. **Figure 2C**. The maximum optomotor responses of rescued *hdc*^*JK*910^ mutants are wild-type-like (OR_rescue_ = 1.9 ± 0.2 a.u., OR_wild-type_ = 2.0 ± 0.2 a.u., *p* = 0.715, *n*_rescue_ = 5, *n*_wild-type_ = 7 flies). **(A–G)** Mean ± SEM, two-tailed *t*-test, unless stated otherwise. **(A,B,D)**: Modulation was the average standard deviation in each response segment, estimated from five consecutive 2 s samples: 11–20 s from each Bright, Mid, and Dim step onward. These averages for each corresponding Dim, Mid and Bright sections were collected across the tested fly populations and compared statistically.

In the first part, we recorded wild-type and *hdc*^*JK*910^ R1–R6 photoreceptor outputs to a repeated naturalistic light intensity time series that intensified and weakened in logarithmic steps as a staircase function (**Figures 9A,B**, respectively). The difference in the recordings showed unambiguously that *hdc*^*JK*910^ R1–R6 photoreceptor output to Mid and Bright stimulation contained less modulation than the wild-type, irrespective of whether the stimuli followed darkness or different brightness modulation (Bright1, Mid1, Dim1, Dim2, Mid2, Bright2). Thus, these data confirmed our observations and analyses for specific stimuli (**Figures 6** and **7**), generalizing their conclusions over a broad dynamic light intensity range. The recordings also confirmed that *hdc*^*JK*910^ photoreceptors are more depolarized than the wild-type in darkness (cf. **Figure 5H**); here, showing ∼5.3 mV higher resting potential (*p* = 0.013) prior light stimulation – in support of them receiving tonic enhanced interneuron feedback.

But most importantly, the recordings enabled us to systematically quantify how the frequency content of the additional modulation in the wild-type photoreceptor output changed during light intensity transitions in naturalistic stimulation. This is shown in **Figure 9C** as the difference in the corresponding power spectra of wild-type and *hdc*^*JK*910^ photoreceptor outputs for the first Bright, Mid, and Dim stimulus sections (cf. **Figures 9A,B**). As predicted for high signal-to-noise ratio light conditions, the modulation added phasic components, seen as a band-passing frequency distribution with the peak at 10 Hz, over *hdc*^*JK*910^photoreceptor output frequency range during Mid and Bright stimulation. However, during low signal-to-noise ratio Dim stimulation, its contribution was much less. Thus, extra modulation in wild-type R1–R6 output comes from interneuron feedback.

In the second part of the experiment, we recorded R1–R6 photoreceptor outputs in the histamine-rescued *hdc*^*JK*910^ mutants (**Figure 9D**). The synaptic feedforward function of photoreceptor-interneuron synapses in *hdc*^*JK*910^ mutants were rescued by feeding them with histamine (Melzig et al., 1998). Consequently, we reasoned, histamine uptake should also recover the interneurons’ feedback modulation to photoreceptor output. Indeed, we found that their voltage responses to the sophisticated staircase light stimulus now closely resembled the corresponding wild-type output (**Figure 9A**; their respective SD changes were similar at all the tested brightness levels: 0.070 ≤*p* ≤ 0.991, two-tailed *t*-test). In line with the feedback hypothesis, their Bright and Mid sections carried characteristic phasic modulation (**Figure 9E**), with broadly comparable stimulus power distributions to the wild-type counterparts (cf. **Figure 9C**). Their dissimilarities mainly reflected noise and natural variability in fewer *hdc*^*JK*910^ recordings (*n* = 3 flies) as the corresponding histamine-rescued *hdc*^*JK*910^ power spectra did not differ statistically from the wild-type up to 100 Hz (*p* >>0.05, two-tailed *t*-test). In further agreement, histamine-rescue also lowered the resting potentials of *hdc*^*JK*910^ photoreceptors to the wild-type level, indicating tonic excitatory interneuron feedback as the likely cause for their initial difference.

Finally, we used the basic test assays to quantify the rescued mutants’ vision. Their ERG (**Figure 9F**) and optomotor responses (**Figure 9G**) approached the wild-type dynamics, indicating that these flies would now see normally, or near-normally; cf. **Figures 2B,C**.

The close correspondence between the experiments (histamine-rescue results) and the theory (predictions of the interneuron feedback hypothesis) allows us to conclude that dynamic network regulation is critical for normal *Drosophila* photoreceptor function and vision.

## Discussion

Eyes must continuously sample information about the world and adapt to its similarities and differences to see well. While facing physical encoding constraints and vast intensity changes in natural environments, network adaptation to prevailing light conditions is expected to improve the eyes’ neural representation of visual scenes (neural images), and so the efficiency and performance of vision (Laughlin, 1981; Brenner et al., 2000; Nikolaev et al., 2009; Zheng et al., 2009). In this study, we have systematically investigated how dynamic network adaptation, which in eye circuits plays a major role in maintaining time-dependent visual capabilities, affects *Drosophila*’s photoreceptor function. This was done by comparing intracellular voltage responses of *hdc*^*JK*910^ photoreceptors, which owing to their blocked feedforward pathway cannot receive dynamic feedback from interneurons, to those of wild-type photoreceptors, which receive normal interneuron feedback. We found that a lack of synaptic feedforward transmission causes both dynamic and homeostatic changes in photoreceptors’ signaling properties and performance, and characterized these changes. Finally, we showed that rescuing photoreceptors’ feedforward pathway restores feedback signals, and consequently photoreceptor function and fly vision returns to normal. Our findings demonstrate the importance of interneuron feedback in regulating the quality of photoreceptor output under changing light conditions and in robustness of vision.

### Excitatory Feedback Hypothesis Predicts *hdc*^*JK*910^ R1–R6s’ Distinctive Response Characteristics

Our results are consistent with the excitatory interneuron feedback hypothesis (Zheng et al., 2006, 2009; Nikolaev et al., 2009) and the lamina interneurons’ neurotransmitter immunohistochemistry (Kolodziejczyk et al., 2008; Raghu and Borst, 2011; Takemura et al., 2011; Hu et al., 2015). Most critically, *hdc*^*JK*910^ photoreceptors’ membrane properties in darkness and responses to bright stimuli indicate that the major interneuron feedback to *Drosophila* photoreceptors cannot be inhibitory. Missing inhibitory feedback would increase modulation in *hdc*^*JK*910^ photoreceptor output, but we see the opposite (**Figures 7–9**). Although feedback inhibition undoubtedly plays an important role in modulating signals within and between neural cartridges, where it mediates lateral inhibition as witnessed by Ca^2+^-imaging at the medulla level (Freifeld et al., 2013), its contribution to shaping time-dependent photoreceptor output seems minute at best.

**FIGURE 10:**
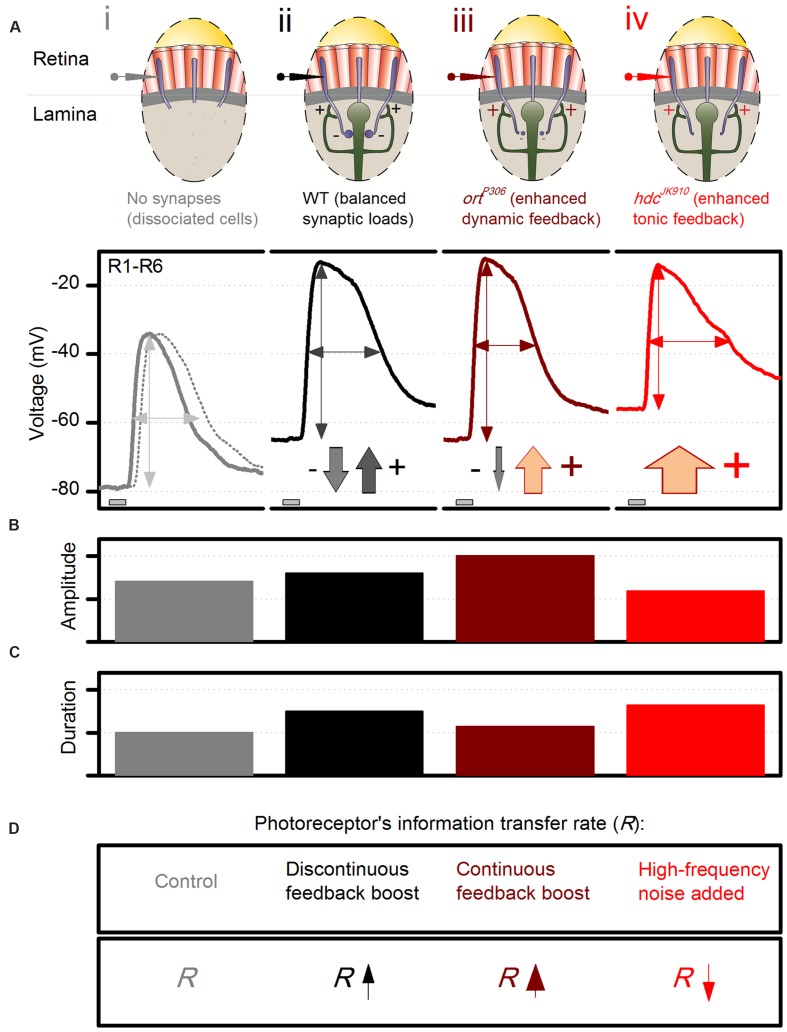
**Dynamic network regulation on photoreceptor function.** Schematic and qualitative representation of how excitatory interneuron feedback shapes voltage responses of dark-adapted R1–R6 photoreceptors to a brief bright pulse. **(A)** Voltage output: (i), without synaptic contacts (WT, continuous line, and *hdc*^*JK*910^, dotted line, of *ex vivo* dissociated photoreceptors); (ii), of wild-type photoreceptors; (iii), of *ort*^*P*306^ photoreceptors, which receive enhanced excitatory dynamic interneuron feedback (modulation); (iv), of *hdc*^*JK*910^ photoreceptors. The down-arrows (inhibitory histaminergic feedforward) and up-arrows (excitatory synaptic feedback from interneurons) indicate their relative contributions to photoreceptor output regulation; e.g., there is only tonic excitatory interneuron feedback to *hdc*^*JK*910^ photoreceptors. **(B)** Effect of different feedback conditions on the response size. **(C)** Effect of different feedback conditions on the response speed. **(D)** Effect of different feedback conditions on the photoreceptors’ information transfer rate, *R*.

To put our findings in the context of network processing, we first illustrate with a schematic photoreceptor output chart (**Figure 10**) how different experimental observations match the key predictions of the excitatory feedback model, at the tested conditions of: (i) abolished synaptic contacts; (ii) normal contacts; (iii) blocked feedforward; and (iv) reduced feedforward. In essence, the model states that *in vivo* R1–R6 photoreceptor output to light changes carries two main components: the phototransduction response and the excitatory feedback response from interneurons.

(i) When photoreceptors are severed from the synaptic network in dissociated ommatidia (**Figure 10A**) and voltage-clamped, their response is the phototransduction response (cf. **Figure 5C**, which shows the corresponding LIC). Its amplitude (**Figure 10B**) and duration (**Figure 10C**) follow adaptive changes in light information sampling by 30,000 microvilli and concurrent membrane filtering (**Figure 10D**; Song et al., 2012; Hardie and Juusola, 2015). Without the depolarizing feedback conductances, the photoreceptors’ resting potentials in darkness settle to low values, as hyperpolarized by their strong intrinsic K^+^-conductances (Hardie, 1991a; Vähäsöyrinki et al., 2006). This was earlier confirmed *in vivo* by continuous intracellular photoreceptor recordings in *shibire*^*TS*1^ mutants (Zheng et al., 2006). Warming them >28°C silenced synaptic transmission between photoreceptors and interneurons. With the feedback ceasing, the photoreceptors swiftly hyperpolarized to 15–20 mV lower potentials than at 18°C, where the feedback functioned normally.
(ii) When photoreceptors are normally engaged in the synaptic network, excitatory interneuron feedback phasically modulates their rising and decaying responses (cf. **Figure 9C**), in particular to bright stimulation (**Figure 10A**). This modulation, which due to pooling six photoreceptor signals in the interneurons (neural superposition) has a higher information content than that of a single photoreceptor (**Figure 10D**; Zheng et al., 2006), accentuates intensity differences in responses over time (**Figures 10B,C**). In darkness, tonic excitatory feedback to photoreceptors strengthens because the interneurons receive less histamine, and so are more depolarized than in light. Hence, photoreceptors’ resting potentials are more depolarized than without the feedback (i).
(iii) When the sign-inverting/hyperpolarizing feedforward transmission from photoreceptors is reduced in a hypomorphic mutant (*ort*^*P*306^) of the postsynaptic histamine receptor, interneurons become more depolarized (Zheng et al., 2006). In return, their modulation releases more excitatory neurotransmitters onto photoreceptor axon terminals than in the wild-type situation (**Figures 10A–D**; iii vs. ii). In *ort*^*P*306^ mutants with weaker feedforward, the enhanced synaptic feedback signals drive photoreceptors to larger responses (**Figure 10C**) with faster kinetics (**Figure 10B**); for example, *ort*^*P*306^ output to the bright pulse peaks and decays ∼40% faster than in the wild-type. The enhanced interneuron feedback also carries abnormal high-frequency modulation (likely resulting from accelerated histamine-receptor kinetics), which enriches photoreceptors’ signal content (Zheng et al., 2006) (**Figure 10D**).
(iv) In *hdc*^*JK*910^ mutants, the completely blocked feedforward pathway probably elevates LMCs and amacrine cells to even higher depolarized levels than those of *ort*^*P*306^ and *ebon*y. Accordingly, *hdc*^*JK*910^ photoreceptors receive excessive excitatory feedback. Unlike in the wild-type (or *ort*^*P*306^), however, this feedback signal lacks modulation and tonically depolarizes *hdc*^*JK*910^ photoreceptors’ resting potentials above the wild-type values (**Figure 10A**; iv vs. ii), as was seen in the recordings (**Figures 5G** and **9B**). Nonetheless, because the *hdc*^*JK*910^ interneurons are effectively blind, their feedback signals cannot improve the quality of photoreceptor output – its amplitude or frequency representations – to light changes (**Figure 10D**).

Additionally, other intrinsic (homeostatic) mechanisms are likely to compensate for these extrinsic changes and thus convert *hdc*^*JK*910^ photoreceptors into a distinctive regime with unique response characteristics (**Figures 10B,C**), rather than mimicking or exacerbating those observed in *ort*^*P*306^ and *ebony* mutants (Zheng et al., 2006). For instance, rebalancing of intrinsic ion channels (Vähäsöyrinki et al., 2006) may restore their membrane input resistance to wild-type levels in darkness (**Figure 5F**), while the cells’ lower membrane capacitance (**Figure 5D**) may accelerate the conduction of their slower macroscopic LICs (**Figure 5C**). Such possible synergistic contributions were evidenced under brief light stimulation by the equally fast rise times of the *hdc*^*JK*910^ and wild-type photoreceptors’ voltage responses to brief light pulses (**Figure 3C**).

### *hdc*^*JK*910^ Photoreceptors’ Compromised Operational Range

Compared to wild-type flies and the synaptic mutants, which have either faulty histamine receptors (*ort*^*P*306^) or histamine recycling (*ebony*; Zheng et al., 2006), the most notable characteristic of *hdc*^*JK*910^ photoreceptors is their reduced sensitivity to bright and prolonged light stimuli. *hdc*^*JK*910^ R1–R6s produced smaller responses to long light pulses (**Figure 4**), with *hdc*^*JK*910^ ERGs consistently showing smaller photoreceptor components (**Figures 2B,C**). Furthermore, the amplitude distribution (or modulation) of their responses contracted during Bright naturalistic stimulation (**Figures 6E,F** and **7A,B**), which accordingly is reflected in their lower signal power spectra (**Figures 7C,D**). With their output beginning to stall at Mid intensities (**Figures 7B** and **9D**), *hdc*^*JK*910^ photoreceptors generated lower information rates to Bright naturalistic stimulation (6/8 cells; **Figures 7G** and **8**). Together, these results imply that *hdc*^*JK*910^ photoreceptors have a narrower operational range: with brightening stimulation, their voltage responses reach maximum amplitude and information transfer rates before wild-type photoreceptors and their encoding performance begins to saturate earlier, because they lack the additional synaptic information component from the network.

### Abnormal Feedback Affects *hdc*^*JK*910^ Photoreceptor Output

We cannot rule out other defects in *hdc*^*JK*910^ phototransduction cascade, which might affect their light-induced responses. However, *ex vivo* properties of mutant photoreceptors cannot explain their *in vivo* characteristics. For example, the slightly lengthier macroscopic LICs and wild-type-like somatic membrane conductances found in dissociated *hdc*^*JK*910^ photoreceptors do not directly result in their *in vivo* counterpart’s contracted responses to long light pulses and naturalistic stimuli.

Therefore, the detrimental features of mutant photoreceptor outputs are largely attributable to the abnormal feedback signals from their interneurons. As demonstrated in (Zheng et al., 2006), feedforward and feedback signals dynamically contribute to photoreceptor and interneuron outputs. When the probability of light saturation is low, the stronger synaptic transmission in both pathways helps to amplify their response amplitudes. Moreover, since each lamina cartridge receives input from six different photoreceptors, which sample light from a small area in space (Meinertzhagen and O’Neil, 1991), the signal-to-noise ratios of L1–L3s’ and probably ACs’ voltage responses are higher than those of photoreceptors’ (Juusola et al., 1995; Zheng et al., 2006). In return, the high quality interneuron feedback – especially during high signal-to-noise ratio stimulation (Bright and Mid) – helps to improve photoreceptor signal quality (Zheng et al., 2006). When depolarizing and hyperpolarizing outputs of photoreceptors and interneurons, respectively, are large, low-frequency synaptic loads should be reduced to prevent signal saturation in exchange of increasing high-frequency synaptic load (phasic signals). Lacking these dynamic mechanisms, along with the artificially high resting potentials (**Figure 5C**), are therefore the probable reasons for the weakened responses observed in *hdc*^*JK*910^ photoreceptors.

The evidence about why the suggested (but never shown) histamine autoreceptors (Hardie et al., 1988) in R1–R6 terminals are unlikely to contribute to these and our previous findings is discussed in (Zheng et al., 2006).

### Histamine-Uptake Rescues *hdc*^*JK*910^ Photoreceptor Output to Normal Wild-Type Dynamics

The theoretical considerations above were strongly supported experimentally by the recovery of photoreceptor function in *hdc*^*JK*910^ mutants, fed on histamine-rich diet, thereby excluding any unforeseen pleiotropic or developmental effects of the *hdc*^*JK*910^ mutation. The rescued *hdc*^*JK*910^ photoreceptors showed normal voltage output with wild-type-like modulation to Bright and Mid naturalistic stimulation. Intriguingly, this recovered modulation carried band-pass frequency distribution, which during with bright stimulation, output power peaked at 10 Hz (**Figure 9E**) – similar to the LMC output’s frequency distribution (Zheng et al., 2009), suggesting that it was largely phasic and came from the interneurons. The histamine-rescue further lowered *hdc*^*JK*910^ photoreceptors’ resting potentials to wild-type values (**Figure 9D**), implying that the probable excitatory overload, which these cells received from interneuron feedback, returned to its normal range when the interneurons started functioning normally (**Figure 9F**), as judged from their ERGs’ normal-like on- and off-transients.

## Conclusion

Photoreceptor voltage output is shaped by a complex interaction between the phototransduction current, voltage-sensitive membrane and synaptic feedback. How photoreceptors receive, process, and transmit information depends upon how these different components interact, and the appropriate balance between them is critical for normal vision. In this article, we showed that lack of synaptic feedforward transmission to visual interneurons in *hdc*^*JK*910^ mutant causes both dynamic and homeostatic changes in *Drosophila* photoreceptors’ signaling properties and performance, and quantified these changes over a broad light intensity range. Our results imply that synaptic feedback to photoreceptors carries mostly excitatory phasic modulation, which neurally accentuates intensity differences in light stimulation, and highlight the general importance of local interneurons as dynamic regulators of photoreceptor function and normal vision.

## Author Contributions

AD, UF, RCH, and MJ designed research; AD, UF, SD, XL, MB, RCH, and MJ performed research; AD, RCH, and MJ analyzed data; AD and MJ wrote the paper.

## Conflict of Interest Statement

The authors declare that the research was conducted in the absence of any commercial or financial relationships that could be construed as a potential conflict of interest.

## References

[B1] Abou Tayoun A. N. Li X. F. Chu B. Hardie R. C. Juusola M. Dolph P. J. (2011). The *Drosophila* SK channel (dSK) contributes to photoreceptor performance by mediating sensitivity control at the first visual network. *J. Neurosci.* 31 13897–13910. 10.1523/Jneurosci.3134-11.2011 21957252 PMC3758547

[B2] Brenner N. Bialek W. van Steveninck R. D. (2000). Adaptive rescaling maximizes information transmission. *Neuron* 26 695–702. 10.1016/S0896-6273(00)81205-2 10896164

[B3] Burg M. G. Sarthy P. V. Koliantz G. Pak W. L. (1993). Genetic and molecular-identification of a *Drosophila* histidine-decarboxylase gene required in photoreceptor transmitter synthesis. *Embo J.* 12 911–919. 8096176 10.1002/j.1460-2075.1993.tb05732.x PMC413291

[B4] Burkhardt D. A. (1993). synaptic feedback, depolarization, and color opponency in cone photoreceptors. *Vis. Neurosci.* 10 981–989. 10.1017/S0952523800010087 8257672

[B5] Coombe P. E. (1986). The large monopolar cells L1 and L2 are responsible for erg transients in *Drosophila*. *J. Compar. Physiol. A-Sens. Neural Behav. Physiol.* 159 655–665. 10.1007/Bf00612038

[B6] Freifeld L. Clark D. A. Schnitzer M. J. Horowitz M. A. Clandinin T. R. (2013). GABAergic lateral interactions tune the early stages of visual processing in *Drosophila*. *Neuron* 78 1075–1089. 10.1016/j.neuron.2013.04.024 23791198 PMC3694283

[B7] Friederich U. Coca D. Billings S. Juusola M. (2009). Data modelling for analysis of adaptive changes in fly photoreceptors. *Neur. Inform. Process. Proc.* 5863 34–48.

[B8] Gonzalez-Bellido P. T. Wardill T. J. Kostyleva R. Meinertzhagen I. A. Juusola M. (2009). Overexpressing temperature-sensitive dynamin decelerates phototransduction and bundles microtubules in *Drosophila* photoreceptors. *J. Neurosci.* 29 14199–14210. 10.1523/Jneurosci.2873-09.2009 19906968 PMC2833399

[B9] Hardie R. C. (1987). Is histamine a neurotransmitter in insect photoreceptors. *J. Compar. Physiol. A-Sens. Neur. Behav. Physiol.* 161 201–213. 10.1007/Bf00615241 2442380

[B10] Hardie R. C. (1989). A histamine-activated chloride channel involved in neurotransmission at a photoreceptor synapse. *Nature* 339 704–706. 10.1038/339704a0 2472552

[B11] Hardie R. C. (1991a). Voltage-sensitive potassium channels in *Drosophila* photoreceptors. *J. Neurosci.* 11 3079–3095. 1941075 10.1523/JNEUROSCI.11-10-03079.1991 PMC6575426

[B12] Hardie R. C. (1991b). Whole-cell recordings of the light-induced current in dissociated *Drosophila* photoreceptors - evidence for feedback by calcium permeating the light-sensitive channels. *Proc. R. Soc. B-Biol. Sci.* 245 203–210. 10.1098/rspb.1991.0110

[B13] Hardie R. C. Juusola M. (2015). Phototransduction in *Drosophila*. *Curr. Opin. Neurobiol.* 34 37–45. 10.1016/j.conb.2015.01.008 25638280

[B14] Hardie R. C. Laughlin S. B. Osorio D. (1988). “Early visual processing in the compound eye: physiology and pharmacology of the retina-lamina projection in the fly,” in *Neurobiology of Sensory Systems*, eds Singh R. Strausfeld N. (New York, NY: Plenum Press), 23–42.

[B15] Hardie R. C. Martin F. Cochrane G. W. Juusola M. Georgiev P. Raghu P. (2002). Molecular basis of amplification in *Drosophila* phototransduction: roles for G protein, phospholipase C, and diacylglycerol kinase. *Neuron* 36 689–701. 10.1016/S0896-6273(02)01048-6 12441057

[B16] Heisenberg M. (1971). Separation of receptor and lamina potentials in electroretinogram of normal and mutant *Drosophila*. *J. Exp. Biol.* 55 85–100. 5001616 10.1242/jeb.55.1.85

[B17] Henderson S. R. Reuss H. Hardie R. C. (2000). Single photon responses in *Drosophila* photoreceptors and their regulation by Ca2+. *J. Physiol. Lon.* 524 179–194. 10.1111/j.1469-7793.2000.00179.x PMC2269851 10747191

[B18] Hu W. Wang T. T. Wang X. Han J. H. (2015). I-h channels control feedback regulation from amacrine cells to photoreceptors. *PLoS Biol.* 13:e1002115 10.1371/journal.pbio.1002115 PMC4382183 25831426

[B19] Jackman S. L. Babai N. Chambers J. J. Thoreson W. B. Kramer R. H. (2011). A positive feedback synapse from retinal horizontal cells to cone photoreceptors. *PLoS Biol.* 9:e1001057 10.1371/journal.pbio.1001057 PMC3086870 21559323

[B20] Juusola M. de Polavieja G. G. (2003). The rate of information transfer of naturalistic stimulation by graded potentials. *J. Gen. Physiol.* 122 191–206. 10.1085/jgp.200308824 12860926 PMC2229540

[B21] Juusola M. Hardie R. C. (2001a). Light adaptation in *Drosophila* photoreceptors: I. Response dynamics and signaling efficiency at 25 degrees C. *J. Gen. Physiol.* 117 3–25. 10.1085/jgp.117.1.3 11134228 PMC2232468

[B22] Juusola M. Hardie R. C. (2001b). Light adaptation in *Drosophila* photoreceptors: II. Rising temperature increases the bandwidth of reliable signaling. *J. Gen. Physiol.* 117 27–41. 10.1085/jgp.117.1.27 11134229 PMC2232470

[B23] Juusola M. Kouvalainen E. Järvilehto M. Weckström M. (1994). Contrast gain, signal-to-noise ratio, and linearity in light-adapted blowfly photoreceptors. *J. Gen. Physiol.* 104 593–621. 10.1085/jgp.104.3.593 7807062 PMC2229225

[B24] Juusola M. Uusitalo R. O. Weckstrom M. (1995). Transfer of graded potentials at the photoreceptor interneuron synapse. *J. Gen. Physiol.* 105 117–148. 10.1085/jgp.105.1.117 7537323 PMC2216927

[B25] Kolodziejczyk A. Sun X. J. Meinertzhagen I. A. Nässel D. R. (2008). Glutamate, GABA and acetylcholine signaling components in the lamina of the *Drosophila* visual system. *PLoS ONE* 3:e2110 10.1371/Journal.Pone.0002110 PMC2373871 18464935

[B26] Laughlin S. (1981). A simple coding procedure enhances a neurons information capacity. *Z. Fur Naturforschung C-A J. Biosci.* 36 910–912. 7303823

[B27] Laughlin S. B. Howard J. Blakeslee B. (1987). Synaptic limitations to contrast coding in the retina of the blowfly *Calliphora*. *Proc. R. Soc. Ser. B Biol. Sci.* 231 437–467. 10.1098/rspb.1987.0054 2892202

[B28] Meinertzhagen I. A. O’Neil S. D. (1991). Synaptic organization of columnar elements in the lamina of the wild-type in *Drosophila*-melanogaster. *J. Compar. Neurol.* 305 232–263. 10.1002/cne.903050206 1902848

[B29] Melzig J. Buchner S. Wiebel F. Wolf R. Burg M. Pak W. L. (1996). Genetic depletion of histamine from the nervous system of *Drosophila* eliminates specific visual and mechanosensory behavior. *J. Compar. Physiol. A-Sen. Neural Behav. Physiol.* 179 763–773. 10.1007/BF00207355 8956497

[B30] Melzig J. Burg M. Gruhn M. Pak W. L. Buchner E. (1998). Selective histamine uptake rescues photo- and mechanoreceptor function of histidine decarboxylase-deficient *Drosophila* mutant. *J. Neurosci.* 18 7160–7166. 9736639 10.1523/JNEUROSCI.18-18-07160.1998 PMC6793226

[B31] Nikolaev A. Zheng L. Wardill T. J. O’Kane C. J. de Polavieja G. G. Juusola M. (2009). Network adaptation improves temporal representation of naturalistic stimuli in *Drosophila* eye: II mechanisms. *PLoS ONE* 4:e4306 10.1371/Journal.Pone.0004306 PMC2628722 19180195

[B32] Raghu S. V. Borst A. (2011). Candidate glutamatergic neurons in the visual system of *Drosophila*. *PLoS ONE* 6:e19472 10.1371/journal.pone.0019472 PMC3088675 21573163

[B33] Reynolds E. S. (1963). Use of lead citrate at high ph as an electron-opaque stain in electron microscopy. *J. Cell Biol.* 17 208–212. 10.1083/Jcb.17.1.208 13986422 PMC2106263

[B34] Rivera-Alba M. Vitaladevuni S. N. Mischenko Y. Lu Z. Y. Takemura S. Y. Scheffer L. (2011). Wiring economy and volume exclusion determine neuronal placement in the *Drosophila* brain. *Curr. Biol.* 21 2000–2005. 10.1016/j.cub.2011.10.022 22119527 PMC3244492

[B35] Sarthy P. V. (1991). Histamine - a neurotransmitter candidate for *Drosophila* photoreceptors. *J. Neurochem.* 57 1757–1768. 10.1111/j.1471-4159.1991.tb06378.x 1717657

[B36] Satoh A. K. Xia H. Yan L. Liu C. H. Hardie R. C. Ready D. F. (2010). Arrestin translocation is stoichiometric to rhodopsin isomerization and accelerated by phototransduction in *Drosophila* photoreceptors. *Neuron* 67 997–1008. 10.1016/j.neuron.2010.08.024 20869596 PMC2946946

[B37] Shannon C. E. (1948). A Mathematical Theory of Communication. *Bell Syst. Tech. J.* 27 623–656. 10.1002/j.1538-7305.1948.tb00917.x

[B38] Shaw S. R. Frohlich A. Meinertzhagen I. A. (1989). Direct connections between the R7/8 and R1-6 photoreceptor subsystems in the dipteran visual-system. *Cell Tissue Res.* 257 295–302. 10.1007/Bf00261833 2776184

[B39] Song Z. Juusola M. (2014). Refractory sampling links efficiency and costs of sensory encoding to stimulus statistics. *J. Neurosci.* 34 7216–7237. 10.1523/Jneurosci.4463-13.2014 24849356 PMC4028498

[B40] Song Z. Postma M. Billings S. A. Coca D. Hardie R. C. Juusola M. (2012). Stochastic, adaptive sampling of information by microvilli in fly photoreceptors. *Curr. Biol.* 22 1371–1380. 10.1016/j.cub.2012.05.047 22704990 PMC3420010

[B41] Sterling P. (1983). Microcircuitry of the cat retina. *Annu. Rev. Neurosci.* 6 149–185. 10.1146/annurev.ne.06.030183.001053 6838139

[B42] Takemura S. Y. Karuppudurai T. Ting C. Y. Lu Z. Y. Lee C. H. Meinertzhagen I. A. (2011). Cholinergic circuits integrate neighboring visual signals in a *Drosophila* motion detection pathway. *Curr. Biol.* 21 2077–2084. 10.1016/j.cub.2011.10.053 22137471 PMC3265035

[B43] Tang S. M. Guo A. (2001). Choice behavior of *Drosophila* facing contradictory visual cues. *Science* 294 1543–1547. 10.1126/science.1058237 11711680

[B44] Thoreson W. B. Babai N. Bartoletti T. M. (2008). Feedback from horizontal cells to rod Photoreceptors in vertebrate retina. *J. Neurosci.* 28 5691–5695. 10.1523/Jneurosci.0403-08.2008 18509030 PMC3552531

[B45] Thoreson W. B. Mangel S. C. (2012). Lateral interactions in the outer retina. *Prog. Retin. Eye Res.* 31 407–441. 10.1016/j.preteyeres.2012.04.003 22580106 PMC3401171

[B46] Uusitalo R. O. Juusola M. Kouvalainen E. Weckstrom M. (1995a). Tonic transmitter release in a graded potential synapse. *J. Neurophysiol.* 74 470–473. 7472349 10.1152/jn.1995.74.1.470

[B47] Uusitalo R. O. Juusola M. Weckstrom M. (1995b). Graded responses and spiking properties of identified first-order visual interneurons of the fly compound eye. *J. Neurophysiol.* 73 1782–1792. 7623079 10.1152/jn.1995.73.5.1782

[B48] Vähäsöyrinki M. Niven J. E. Hardie R. C. Weckström M. Juusola M. (2006). Robustness of neural coding in *Drosophila* photoreceptors in the absence of slow delayed rectifier K+ channels. *J. Neurosci.* 26 2652–2660. 10.1523/Jneurosci.3316-05.2006 16525044 PMC6675149

[B49] van Hateren J. H. (1997). Processing of natural time series of intensities by the visual system of the blowfly. *Vision Res.* 37 3407–3416. 10.1016/S0042-6989(97)00105-3 9425553

[B50] van Hateren J. H. Snippe H. P. (2001). Information theoretical evaluation of parametric models of gain control in blowfly photoreceptor cells. *Vision Res.* 41 1851–1865. 10.1016/S0042-6989(01)00052-9 11369048

[B51] Wardill T. J. List O. Li X. F. Dongre S. McCulloch M. Ting C. Y. (2012). Multiple spectral inputs improve motion discrimination in the *Drosophila* visual system. *Science* 336 925–931. 10.1126/science.1215317 22605779 PMC6528803

[B52] Zheng L. Nikolaev A. Wardill T. J. O’Kane C. J. de Polavieja G. G. Juusola M. (2009). Network adaptation improves temporal representation of naturalistic stimuli in *Drosophila* eye: I dynamics. *PLoS ONE* 4:e4307 10.1371/Journal.Pone.0004307 PMC2628724 19180196

[B53] Zheng L. Polavieja G. G. Wolfram V. Asyali M. H. Hardie R. C. Juusola M. (2006). Feedback network controls photoreceptor output at the layer of first visual synapses in *Drosophila*. *J. Gen. Physiol.* 127 495–510. 10.1085/jgp.200509470 16636201 PMC2151524

